# Supplementary material for: Association between parental psychiatric conditions and offspring psychiatric, behavioral, and psychosocial outcomes: A Swedish population-based children-of-monozygotic twins study
Source: PLoS Med. 2025 Oct 21;22(10):e1004784. doi: 10.1371/journal.pmed.1004784 (PMC12571287; doi:10.1371/journal.pmed.1004784)
Supplement: S1 Checklist — (DOC) [file pmed.1004784.s001.doc]

STROBE Statement—Checklist of items that should be included in reports of ***cohort studies***

|  | Item No | Recommendation |
| --- | --- | --- |
| **Title and abstract** | 1 | (*a*) Indicate the study’s design with a commonly used term in the title or the abstract **[Reported in Abstract]** |
|  |  | (*b*) Provide in the abstract an informative and balanced summary of what was done and what was found **[Reported in Abstract]** |
| Introduction | | |
| Background/rationale | 2 | Explain the scientific background and rationale for the investigation being reported **[Reported in Introduction]** |
| Objectives | 3 | State specific objectives, including any prespecified hypotheses **[Reported in Introduction paragraph 7]** |
| Methods | | |
| Study design | 4 | Present key elements of study design early in the paper **[Reported in Introduction paragraphs 3 and 7, in Methods (Study population), and in Figure 1]** |
| Setting | 5 | Describe the setting, locations, and relevant dates, including periods of recruitment, exposure, follow-up, and data collection **[Reported in Methods (Study population)]** |
| Participants | 6 | (*a*) Give the eligibility criteria, and the sources and methods of selection of participants. Describe methods of follow-up **[Reported in Methods (Study population, Exposure)]** |
|  |  | (*b*) For matched studies, give matching criteria and number of exposed and unexposed **[N/A]** |
| Variables | 7 | Clearly define all outcomes, exposures, predictors, potential confounders, and effect modifiers. Give diagnostic criteria, if applicable **[Reported in Methods (Exposure, Outcome, Covariates)]** |
| Data sources/ measurement | 8* | For each variable of interest, give sources of data and details of methods of assessment (measurement). Describe comparability of assessment methods if there is more than one group **[Reported in Methods (Study population, Exposure, Outcome, Covariates) and in S1 Appendix Table A, Table B]** |
| Bias | 9 | Describe any efforts to address potential sources of bias **[Reported in Discussion paragraph 4 and in Limitation]** |
| Study size | 10 | Explain how the study size was arrived at **[N/A]** |
| Quantitative variables | 11 | Explain how quantitative variables were handled in the analyses. If applicable, describe which groupings were chosen and why **[Reported in Methods (Exposure, Outcome, Covariates) and in Table 1]** |
| Statistical methods | 12 | (*a*) Describe all statistical methods, including those used to control for confounding **[Reported in Methods (Statistical analyses)]** |
|  |  | (*b*) Describe any methods used to examine subgroups and interactions **[Reported in Methods (Sensitivity analyses)]** |
|  |  | (*c*) Explain how missing data were addressed **[Reported in Methods (Study population)]** |
|  |  | (*d*) If applicable, explain how loss to follow-up was addressed **[N/A]** |
|  |  | (*e*) Describe any sensitivity analyses **[Reported in Methods (Sensitivity analyses)]** |
| Results | | |
| Participants | 13* | (a) Report numbers of individuals at each stage of study—eg numbers potentially eligible, examined for eligibility, confirmed eligible, included in the study, completing follow-up, and analysed **[Reported in Result paragraph 1]** |
|  |  | (b) Give reasons for non-participation at each stage **[N/A]** |
|  |  | (c) Consider use of a flow diagram **[N/A]** |
| Descriptive data | 14* | (a) Give characteristics of study participants (eg demographic, clinical, social) and information on exposures and potential confounders **[Reported in Result paragraph 1 and Table 1]** |
|  |  | (b) Indicate number of participants with missing data for each variable of interest **[N/A]** |
|  |  | (c) Summarise follow-up time (eg, average and total amount) **[N/A]** |
| Outcome data | 15* | Report numbers of outcome events or summary measures over time **[Reported in Result paragraph 1 and Table 2]** |
| Main results | 16 | (*a*) Give unadjusted estimates and, if applicable, confounder-adjusted estimates and their precision (eg, 95% confidence interval). Make clear which confounders were adjusted for and why they were included **[Reported in Result]** |
|  |  | (*b*) Report category boundaries when continuous variables were categorized **[Reported in Methods (Outcome) and in S1 Appendix Table A]** |
|  |  | (*c*) If relevant, consider translating estimates of relative risk into absolute risk for a meaningful time period **[N/A]** |
| Other analyses | 17 | Report other analyses done—eg analyses of subgroups and interactions, and sensitivity analyses **[Reported in Result (Sensitivity analyses results)]** |
| Discussion | | |
| Key results | 18 | Summarise key results with reference to study objectives **[Reported in Discussion Paragraph 1]** |
| Limitations | 19 | Discuss limitations of the study, taking into account sources of potential bias or imprecision. Discuss both direction and magnitude of any potential bias **[Reported in Limitation]** |
| Interpretation | 20 | Give a cautious overall interpretation of results considering objectives, limitations, multiplicity of analyses, results from similar studies, and other relevant evidence **[Reported in Discussion]** |
| Generalisability | 21 | Discuss the generalisability (external validity) of the study results **[Reported in Limitation]** |
| Other information | | |
| Funding | 22 | Give the source of funding and the role of the funders for the present study and, if applicable, for the original study on which the present article is based **[Reported in Funding/Support, Role of the Funder/Sponsor]** |

*Give information separately for exposed and unexposed groups.

**Note:** An Explanation and Elaboration article discusses each checklist item and gives methodological background and published examples of transparent reporting. The STROBE checklist is best used in conjunction with this article (freely available on the Web sites of PLoS Medicine at http://www.plosmedicine.org/, Annals of Internal Medicine at http://www.annals.org/, and Epidemiology at http://www.epidem.com/). Information on the STROBE Initiative is available at http://www.strobe-statement.org.
