# Supplementary material for: Association between parental psychiatric conditions and offspring psychiatric, behavioral, and psychosocial outcomes: A Swedish population-based children-of-monozygotic twins study
Source: PLoS Med. 2025 Oct 21;22(10):e1004784. doi: 10.1371/journal.pmed.1004784 (PMC12571287; doi:10.1371/journal.pmed.1004784)
Supplement: S1 Appendix — Table A. Description of registries and variables extracted. Table B. The ICD/ATC code, classified convictions for violent crimes, and the cut-off age for exposures and outcomes. Table C. Association between parental psychiatric conditions and offspring outcomes, HR/OR (95% CI). Table D. Association between parental psychiatric conditions and offspring outcomes in the adjusted within-twin-family model, HR/OR (95% CI), sensitivity analyses. Table E. Association between parental psychiatric conditions and each specific offspring outcome in the adjusted within-twin-family model, HR (95% CI). (DOCX) [file pmed.1004784.s002.docx]

**﻿** **S1 Appendix**

Zhou et al. Association between Parental Psychiatric Conditions and Offspring Psychiatric, Behavioral, and Psychosocial Outcomes

[Table A. Description of registries and variables extracted 2](#_Toc201762686)

[Table B. The ICD/ATC code, classified convictions for violent crimes, and the cut-off age for exposures and outcomes 3](#_Toc201762687)

[Table C. Association between parental psychiatric conditions and offspring outcomes, ﻿HR/OR (95% CI) 5](#_Toc201762688)

[Table D. Association between parental psychiatric conditions and offspring outcomes in the adjusted within-twin-family model, ﻿HR/OR (95% CI), sensitivity analyses 6](#_Toc201762689)

[Table D. Association between parental psychiatric conditions and offspring outcomes in the adjusted within-twin-family model, ﻿HR/OR (95% CI), sensitivity analyses (continued) 7](#_Toc201762690)

[Table E. Association between parental psychiatric conditions and each specific offspring outcome in the adjusted within-twin-family model, ﻿HR (95% CI) 9](#_Toc201762691)

# **Table A. Description of registries and variables extracted**

| **Register** | **Description** | **Variables** |
| --- | --- | --- |
| Total Population Register | Established in 1968 and includes demographic information (e.g., sex, age, place of birth) for the entire Swedish population.^1^ | Individual identification number, birthyear, sex |
| Multi-Generation Register | Links all index persons born in Sweden since 1932 and alive in 1960 to their biological parent.^2^ | Individual identification number |
| National Patient Register | Includes individual-based records of psychiatric inpatient care since 1973 (complete since 1987) and psychiatric outpatient care since 2001 (complete since 2010). All diagnoses were recorded according to the International Classification of Diseases (ICD); 8th (1973/1969-1986), 9th (1987-1996), and 10th (1997-) revision.^3,4^ | Disorders, suicide attempts, violent victimization |
| Prescribed Drug Register | Established in 2005 and contains all dispensed prescribed pharmaceuticals.^5^ | Prescription of medication |
| National Crime Register | Comprises all registered criminal convictions of those aged 15 and older (the age of criminal responsibility) since 1973.^6^ | Court convictions of violent crimes |
| National School Register | Includes averaged junior high final grades (age 15) and (in)eligibility for high school since 1988. | Low school grade |
| Cause of Death Register | Records all deaths in Sweden since 1952 and provides information on causes of death according to ICD.^7^ | Death by suicide |
| Longitudinal Integration Database for Health Insurance and Market Studies | Information from the labor market and educational and social sectors for all individuals registered in Sweden over 16 years of age since 1990.^8^ | Unemployment, attained educational level |
| Swedish Twin Register | Includes nearly all twin pairs born in Sweden from 1886 through 2000.^9^ |  |

# **Table B. The ICD/ATC code, classified convictions for violent crimes, and the cut-off age for exposures and outcomes**

| **Exposure/outcome** | | | **ICD 08 (1969-1986)** | | **ICD 09 (1987-1996)** | | | **ICD 10 (1997-)** | | | | **ATC code** | **Minimum age** | |
| --- | --- | --- | --- | --- | --- | --- | --- | --- | --- | --- | --- | --- | --- | --- |
| Schizophrenia | 295 (except 295.5) | | | | | 295 (except 295F) | | | F20, F25 |  | | | 15 | |
| Bipolar disorder | | | 296.1, 296.3, 296.8, 296.9 | | 296A, 296C, 296D, 296E, 296W, 296X | | | F30, F31 | | | |  | 15 | |
| Depression | | | 300.4 | | 296B, 311, 300E | | | F32, F33 | | | |  | 10 | |
| Anxiety | | | 300 (except 300.3, 300.4) | | 300, (except 300D, 300E) | | | F40, F41 | | | |  | 10 | |
| Obsessive-compulsive disorder | | | 300.3 | | 300D | | | F42 | | | |  | 5 | |
| Post-traumatic stress disorder | | | 307.99 | | 308, 309 | | | F43 | | | |  | 2 | |
| Alcohol-related disorders | | | 291, 303 | | 291, 303, 305A | | | F10 (except F10.5) | | | |  | 12 | |
| Drug-related disorders | | | 304 | | 292, 304, 305X | | | F11-F19 (except F17, F1x.5) | | | |  | 12 | |
| Attention-Deficit/Hyperactivity Disorder | | | - | | 314 | | | F90 | | | |  | 3 | |
| Autism spectrum disorder | | | - | | 299A | | | F84.0, F84.1, F84.5 | | | |  | 2 | |
| Tic disorder | | | 306.2 | | 307C | | | F95 | | | |  | 3 | |
| Learning disorders | | | - | | 315A, 315B | | | F81 | | | |  | 3 | |
| Intellectual disability | | | 311-315 | | 317-319 | | | F70-F79 | | | |  | 2 | |
| Oppositional defiant disorder | | | - | | 312X | | | F91 | | | |  | 3 | |
| Eating disorders | | 784.00 | | 307B, 307F | | | F50 | | | |  | | | 10 |
| Suicide (Suicide attempts and death by suicide) | | | E950-959 E980-989 | | E950-959 E980-989 | | | X60-X84, Y10-Y34 | | | |  | 10 | |
| Anti-alcohol medication | | |  | |  | | |  | | | | N07BB | 12 | |
| Antidepressants | | |  | |  | | |  | | | | N06A | 10 | |
| Antiepileptics | | |  | |  | | |  | | | | N03A | 3 | |
| Anti-opioid medication | | |  | |  | | |  | | | | N07BC | 12 | |
| Antipsychotics | | |  | |  | | |  | | | | N05AA-N05AL, N05AX | 15 | |
| Anxiolytics | | |  | |  | | |  | | | | N05B | 10 | |
| Lithium | | |  | |  | | |  | | | | N05AN | 10 | |
| Stimulants | | |  | |  | | |  | | | | N06B | 3 | |
| Violent crimes | | | homicide (Ch 3, §1-3); assault (Ch 3, §5-6); robbery (Ch 8, §5-6); threats and violence against an officer (Ch 17, §1-2); gross violation of a person’s/woman’s integrity (Ch 4, §4a); unlawful coercion (Ch 4, §4); unlawful threats (Ch 4, §5); kidnapping (Ch 4, §1); illegal confinement (Ch 4, §2); arson (Ch 13, §1-2); intimidation (Ch 4, §7); sexual offence (excluding prostitution and the buying of sexual services but including child pornography) (Ch 6 §1-10, §10A, §12) | | | | | | | | |  | 15 | |
| Poor school performance | | | Defined as ranking in the lowest quintile on the junior high school grade point average, or attaining only a compulsory education level | | | | | | | | |  | 10 | |
| Long-term unemployment | | | Defined as at least one consecutive year unemployment | | | | | | | | |  | 18 | |

# **Table C. Association between parental psychiatric conditions and offspring outcomes, ﻿HR/OR (95% CI)**

| **Exposure** | **Offspring outcomes** | **Between-family model^a^** | | **Within-twin-family model^b^** | |
| --- | --- | --- | --- | --- | --- |
|  |  | **HR/OR (95%CI)** | **P values** | **HR/OR (95%CI)** | **P values** |
| Any parental psychiatric conditions | Any psychiatric conditions | **1.64 (1.51, 1.79)** | <0.001 | **1.28 (1.13, 1.44)** | <0.001 |
|  | Psychotic conditions | **1.83 (1.59, 2.11)** | <0.001 | 1.23 (0.98, 1.55) | 0.072 |
|  | Neurodevelopmental conditions | **1.66 (1.43, 1.93)** | <0.001 | 1.07 (0.87, 1.32) | 0.531 |
|  | Internalizing conditions | **1.51 (1.41, 1.62)** | <0.001 | **1.25 (1.14, 1.38)** | <0.001 |
|  | Substance use disorders | **1.72 (1.47, 2.02)** | <0.001 | 1.27 (1.02, 1.59) | 0.036 |
|  | Externalizing behaviors | **1.56 (1.27, 1.93)** | <0.001 | 1.05 (0.79, 1.41) | 0.722 |
|  | Suicide | **1.60 (1.33, 1.92)** | <0.001 | 1.04 (0.83, 1.30) | 0.729 |
|  | Poor school performance | **1.46 (1.32, 1.62)** | <0.001 | 1.18 (0.97, 1.44) | 0.098 |
|  | Long-term unemployment | **1.47 (1.32, 1.63)** | <0.001 | 1.17 (0.97, 1.42) | 0.109 |
| Parental internalizing conditions | Any psychiatric conditions | **1.72 (1.55, 1.91)** | <0.001 | **1.26 (1.09, 1.45)** | 0.002 |
|  | Psychotic conditions | **2.03 (1.73, 2.38)** | <0.001 | 1.30 (1.01, 1.67) | 0.040 |
|  | Neurodevelopmental conditions | **1.70 (1.44, 2.02)** | <0.001 | 1.07 (0.85, 1.35) | 0.544 |
|  | Internalizing conditions | **1.56 (1.44, 1.70)** | <0.001 | **1.17 (1.04, 1.31)** | 0.009 |
|  | Substance use disorders | **1.56 (1.29, 1.89)** | <0.001 | 1.21 (0.93, 1.57) | 0.163 |
|  | Externalizing behaviors | **1.36 (1.07, 1.72)** | 0.012 | 0.89 (0.62, 1.28) | 0.526 |
|  | Suicide | **1.75 (1.42, 2.17)** | <0.001 | 1.08 (0.82, 1.41) | 0.579 |
|  | Poor school performance | **1.47 (1.30, 1.66)** | <0.001 | **1.35 (1.07, 1.71)** | 0.010 |
|  | Long-term unemployment | **1.33 (1.17, 1.52)** | <0.001 | 1.20 (0.95, 1.50) | 0.121 |
| Parental externalizing conditions | Any psychiatric conditions | **1.56 (1.39, 1.76)** | <0.001 | **1.27 (1.08, 1.51)** | 0.005 |
|  | Psychotic conditions | **1.54 (1.26, 1.88)** | <0.001 | 0.81 (0.59, 1.12) | 0.207 |
|  | Neurodevelopmental conditions | **1.80 (1.48, 2.19)** | <0.001 | **1.45 (1.09, 1.93)** | 0.010 |
|  | Internalizing conditions | **1.34 (1.21, 1.49)** | <0.001 | 0.95 (0.82, 1.10) | 0.481 |
|  | Substance use disorders | **2.19 (1.81, 2.64)** | <0.001 | 1.25 (0.95, 1.64) | 0.108 |
|  | Externalizing behaviors | **2.53 (1.96, 3.26)** | <0.001 | 1.27 (0.93, 1.72) | 0.128 |
|  | Suicide | **1.56 (1.18, 2.05)** | 0.002 | 1.02 (0.71, 1.48) | 0.898 |
|  | Poor school performance | **1.87 (1.63, 2.14)** | <0.001 | 0.99 (0.74, 1.32) | 0.919 |
|  | Long-term unemployment | **1.73 (1.49, 2.01)** | <0.001 | 1.07 (0.80, 1.42) | 0.663 |

Note: ^a^ The sample size of the between-family model is 15,603 unique individuals; ^b^ The sample size of the within-twin-family model is 15,620 individuals as some individuals whose mother and father are both monozygotic twins were included twice. We performed the Cox regression and logisitic regression (depending on the outcome) to estimate the between-family effects and stratified Cox regression and conditional logistic regression to estimate the within-twin-family effects. Adjusted covariates include the highest parental educational level, any partner psychiatric conditions, maternal and paternal age of childbirth, offspring birth year, and sex. The estimator is the odds ratio for poor school performance and long-term unemployment. Estimators shown in bold meet the criteria for Benjamini-Hochberg False Discovery Rate statistical significance (was conducted separately for between-family model [N = 9*3] and within-twin family model [N = 9*3]).

# **Table D.** **Association between parental psychiatric conditions and offspring outcomes in the adjusted within-twin-family model, ﻿**

# **HR/OR (95% CI), sensitivity analyses**

| **Exposure** | **Offspring outcomes** | **Sensitivity 1** | | **Sensitivity 2** | | **Sensitivity 3** | |
| --- | --- | --- | --- | --- | --- | --- | --- |
|  |  | **HR/OR (95%CI)** | **P values** | **HR/OR (95%CI)** | **P values** | **HR/OR (95%CI)** | **P values** |
| Any parental psychiatric conditions | Any psychiatric conditions | **1.50 (1.23, 1.84)** | <0.001 | **1.27 (1.12, 1.45)** | <0.001 | **1.26 (1.06, 1.51)** | 0.010 |
|  | Psychotic conditions | 1.38 (0.95, 2.01) | 0.093 | 1.25 (0.99, 1.58) | 0.064 | 1.25 (0.91, 1.72) | 0.176 |
|  | Neurodevelopmental conditions | **1.63 (1.21, 2.20)** | 0.001 | 1.02 (0.82, 1.27) | 0.876 | 1.06 (0.78, 1.44) | 0.696 |
|  | Internalizing conditions | 1.24 (1.04, 1.47) | 0.015 | **1.27 (1.15, 1.41)** | <0.001 | **1.25 (1.09, 1.44)** | 0.002 |
|  | Substance use disorders | 1.26 (0.88, 1.81) | 0.212 | **1.36 (1.08, 1.70)** | 0.009 | 1.18 (0.86, 1.63) | 0.301 |
|  | Externalizing behaviors | 0.91 (0.58, 1.44) | 0.698 | 1.12 (0.83, 1.52) | 0.450 | 0.91 (0.59, 1.39) | 0.659 |
|  | Suicide | 0.92 (0.64, 1.31) | 0.643 | 1.09 (0.87, 1.38) | 0.441 | 1.03 (0.74, 1.43) | 0.867 |
|  | Poor school performance | 1.28 (0.92, 1.79) | 0.148 | **1.30 (1.06, 1.61)** | 0.014 | 1.15 (0.99, 1.33) | 0.061 |
|  | Long-term unemployment | 1.05 (0.72, 1.53) | 0.812 | 1.16 (0.96, 1.41) | 0.129 | **1.27 (1.10, 1.47)** | 0.001 |
| Parental internalizing conditions | Any psychiatric conditions | **1.57 (1.26, 1.95)** | <0.001 | **1.23 (1.06, 1.42)** | 0.006 | 1.21 (0.99, 1.48) | 0.060 |
|  | Psychotic conditions | 1.68 (1.09, 2.57) | 0.018 | 1.28 (0.99, 1.65) | 0.063 | 1.24 (0.86, 1.77) | 0.246 |
|  | Neurodevelopmental conditions | **1.53 (1.14, 2.05)** | 0.005 | 1.02 (0.81, 1.29) | 0.861 | 1.04 (0.75, 1.43) | 0.829 |
|  | Internalizing conditions | 1.20 (1.01, 1.44) | 0.038 | **1.16 (1.03, 1.31)** | 0.014 | 1.20 (1.02, 1.42) | 0.030 |
|  | Substance use disorders | 1.19 (0.81, 1.73) | 0.376 | 1.24 (0.95, 1.61) | 0.111 | 1.07 (0.74, 1.54) | 0.733 |
|  | Externalizing behaviors | 0.87 (0.47, 1.60) | 0.645 | 0.99 (0.68, 1.46) | 0.977 | 0.91 (0.53, 1.59) | 0.748 |
|  | Suicide | 1.00 (0.69, 1.45) | 0.996 | 1.13 (0.86, 1.48) | 0.393 | 1.18 (0.81, 1.73) | 0.390 |
|  | Poor school performance | 1.56 (1.08, 2.24) | 0.018 | **1.49 (1.16, 1.90)** | 0.002 | **1.35 (1.15, 1.60)** | <0.001 |
|  | Long-term unemployment | 1.34 (0.88, 2.04) | 0.171 | 1.18 (0.93, 1.48) | 0.168 | **1.30 (1.10, 1.53)** | 0.002 |
| Parental externalizing conditions | Any psychiatric conditions | 1.10 (0.88, 1.38) | 0.395 | **1.41 (1.18, 1.68)** | <0.001 | 1.21 (0.94, 1.56) | 0.145 |
|  | Psychotic conditions | 0.76 (0.51, 1.13) | 0.171 | 0.88 (0.61, 1.28) | 0.508 | 0.92 (0.59, 1.43) | 0.705 |
|  | Neurodevelopmental conditions | 1.47 (1.06, 2.03) | 0.019 | **2.01 (1.43, 2.82)** | <0.001 | **1.77 (1.16, 2.72)** | 0.008 |
|  | Internalizing conditions | 0.93 (0.76, 1.15) | 0.516 | 0.97 (0.83, 1.14) | 0.701 | 0.93 (0.74, 1.16) | 0.522 |
|  | Substance use disorders | 1.09 (0.77, 1.54) | 0.635 | **1.44 (1.07, 1.95)** | 0.017 | 1.01 (0.66, 1.56) | 0.952 |
|  | Externalizing behaviors | 1.39 (0.96, 2.04) | 0.084 | 1.08 (0.76, 1.53) | 0.668 | 1.06 (0.68, 1.65) | 0.801 |
|  | Suicide | 0.90 (0.52, 1.57) | 0.711 | 1.46 (1.04, 2.05) | 0.027 | 1.02 (0.59, 1.77) | 0.935 |
|  | Poor school performance | 1.15 (0.79, 1.69) | 0.465 | 1.01 (0.73, 1.42) | 0.933 | 0.95 (0.77, 1.17) | 0.616 |
|  | Long-term unemployment | 1.04 (0.69, 1.57) | 0.840 | 1.07 (0.78, 1.45) | 0.680 | 1.12 (0.90, 1.39) | 0.308 |

# **Table D. Association between parental psychiatric conditions and offspring outcomes in the adjusted within-twin-family model, ﻿**

# **HR/OR (95% CI), sensitivity analyses (continued)**

| **Exposure** | **Offspring outcomes** | **Sensitivity 4**^a1^ | | **Sensitivity 4**^a2^ | | **Sensitivity 4**^a3^ | |
| --- | --- | --- | --- | --- | --- | --- | --- |
|  |  | **HR/OR (95%CI)** | **P values** | **HR/OR (95%CI)** | **P values** | **HR/OR (95%CI)** | **P values** |
| Any parental psychiatric conditions | Any psychiatric conditions | 1.09 (0.86, 1.40) | 0.468 | 1.13 (0.89, 1.43) | 0.304 | **1.48 (1.17, 1.87)** | 0.001 |
|  | Psychotic conditions | 1.13 (0.77, 1.65) | 0.548 | 1.03 (0.69, 1.52) | 0.891 | 1.88 (1.14, 3.11) | 0.014 |
|  | Neurodevelopmental conditions | 0.80 (0.47, 1.38) | 0.424 | 0.90 (0.55, 1.46) | 0.665 | 1.53 (1.12, 2.10) | 0.008 |
|  | Internalizing conditions | 1.24 (1.03, 1.48) | 0.020 | 1.27 (1.06, 1.52) | 0.010 | 1.34 (1.08, 1.65) | 0.007 |
|  | Substance use disorders | 1.24 (0.76, 2.02) | 0.383 | 1.05 (0.70, 1.55) | 0.827 | 1.55 (0.98, 2.45) | 0.061 |
|  | Externalizing behaviors | 1.59 (0.90, 2.81) | 0.108 | 0.75 (0.45, 1.27) | 0.286 | 1.04 (0.55, 1.96) | 0.903 |
|  | Suicide | 1.77 (1.12, 2.78) | 0.014 | 1.08 (0.74, 1.59) | 0.679 | **0.45 (0.28, 0.72)** | 0.001 |
|  | Poor school performance | 1.26 (0.87, 1.81) | 0.221 | 1.52 (1.00, 2.31) | 0.049 | 1.28 (0.84, 1.96) | 0.246 |
|  | Long-term unemployment | 1.16 (0.89, 1.53) | 0.273 | 1.27 (0.85, 1.88) | 0.239 | 1.43 (0.68, 3.03) | 0.348 |
| Parental internalizing conditions | Any psychiatric conditions | 0.86 (0.65, 1.13) | 0.272 | 1.20 (0.92, 1.57) | 0.184 | **1.55 (1.19, 2.01)** | 0.001 |
|  | Psychotic conditions | 0.86 (0.54, 1.35) | 0.509 | 1.35 (0.87, 2.08) | 0.180 | 1.62 (0.94, 2.79) | 0.080 |
|  | Neurodevelopmental conditions | 0.71 (0.39, 1.31) | 0.274 | 1.23 (0.75, 2.02) | 0.418 | 1.32 (0.94, 1.83) | 0.106 |
|  | Internalizing conditions | 1.07 (0.87, 1.33) | 0.517 | 1.25 (1.00, 1.56) | 0.049 | 1.37 (1.09, 1.73) | 0.008 |
|  | Substance use disorders | 1.24 (0.72, 2.12) | 0.436 | 0.98 (0.64, 1.49) | 0.924 | 1.28 (0.78, 2.12) | 0.327 |
|  | Externalizing behaviors | 2.58 (1.03, 6.48) | 0.044 | 0.97 (0.52, 1.84) | 0.936 | 0.52 (0.26, 1.04) | 0.065 |
|  | Suicide | **2.24 (1.34, 3.75)** | 0.002 | 1.34 (0.89, 2.03) | 0.166 | 0.59 (0.36, 0.97) | 0.036 |
|  | Poor school performance | 1.55 (0.98, 2.46) | 0.059 | 1.40 (0.88, 2.23) | 0.154 | 1.44 (0.91, 2.28) | 0.122 |
|  | Long-term unemployment | 1.20 (0.85, 1.68) | 0.301 | 1.51 (0.98, 2.33) | 0.060 | 1.52 (0.68, 3.38) | 0.304 |
| Parental externalizing conditions | Any psychiatric conditions | **1.96 (1.31, 2.93)** | 0.001 | 1.01 (0.72, 1.42) | 0.945 | 1.23 (0.90, 1.70) | 0.193 |
|  | Psychotic conditions | 1.59 (0.83, 3.03) | 0.162 | 0.94 (0.58, 1.53) | 0.802 | 0.54 (0.25, 1.17) | 0.119 |
|  | Neurodevelopmental conditions | 1.97 (0.79, 4.88) | 0.144 | 1.61 (0.88, 2.94) | 0.121 | 1.72 (1.08, 2.74) | 0.023 |
|  | Internalizing conditions | 1.08 (0.82, 1.42) | 0.567 | 0.80 (0.62, 1.04) | 0.095 | 1.00 (0.74, 1.35) | 0.990 |
|  | Substance use disorders | 1.08 (0.60, 1.93) | 0.807 | 0.91 (0.52, 1.58) | 0.744 | 1.25 (0.71, 2.23) | 0.440 |
|  | Externalizing behaviors | 1.52 (0.87, 2.67) | 0.145 | 0.78 (0.40, 1.50) | 0.458 | 2.55 (1.27, 5.14) | 0.009 |
|  | Suicide | 1.37 (0.75, 2.50) | 0.309 | 0.67 (0.37, 1.22) | 0.188 | 1.64 (0.53, 5.10) | 0.395 |
|  | Poor school performance | 0.88 (0.48, 1.59) | 0.664 | 1.25 (0.71, 2.21) | 0.439 | 1.35 (0.74, 2.47) | 0.324 |
|  | Long-term unemployment | 1.24 (0.79, 1.94) | 0.349 | 0.78 (0.45, 1.34) | 0.369 | 2.42 (0.83, 7.03) | 0.105 |

Note: Sensitivity 1: only including parents whose first psychiatric conditions were diagnosed before the child at age 18; the sample sizes for any parental psychiatric conditions, parental internalizing conditions, and parental externalizing conditions are 13,920, 14,611, and 15,107, respectively. Sensitivity 2: only including parents whose first psychiatric conditions were diagnosed after childbirth; the sample sizes for any parental psychiatric conditions, parental internalizing conditions, and parental externalizing conditions are 15,402, 15,503, and 15,317, respectively. Sensitivity 3: restricting the age difference to 8 years or less between cousins; the sample size is 14,343 unique individuals (13,675 cousin pairs); we transferred the data structure (cousin clusters) to cousin pairs because we need to compare the age difference between cousin pairs. Sensitivity 4: dividing the study population into three birth cohorts, ^a1^1970-1980, ^a2^1980-1990, ^a3^1990-2000, the sample sizes of the three cohorts are 5,088, 5,328, 5,187, respectively. We performed stratified Cox regression and conditional logistic regression (depending on the outcome) to estimate the within-twin-family effects. All four sensitivity analyses adjusted for the highest parental educational level, any partner psychiatric conditions, maternal and paternal age of childbirth, offspring birth year, and sex. The estimator is the odds ratio for poor school performance and long-term unemployment. Estimators shown in bold meet the criteria for Benjamini-Hochberg False Discovery Rate statistical significance (was conducted separately for each sensitivity analysis).

# **Table E.** **Association between parental psychiatric conditions and each specific offspring outcome in the adjusted within-twin-family model, ﻿HR (95% CI)**

|  |  |  | **﻿Twin parent exposure** | | | | | | | |  |
| --- | --- | --- | --- | --- | --- | --- | --- | --- | --- | --- | --- |
|  |  | **Any parental psychiatric conditions** | | | | **Parental** **internalizing conditions** | | | **Parental externalizing conditions** | | |
| **Offspring outcomes** | | **Sample size^a^** | | **HR (95%CI)** | **P values** | **Sample size^a^** | **HR (95%CI)** | **P values** | **Sample size****^a^** | **HR (95%CI)** | **P values** |
| Psychotic conditions | Schizophrenia | 61 (15) | | 1.00 (0.28, 3.58) | 0.996 | 41 (10) | 1.42 (0.32, 6.33) | 0.647 | 19 (4) | 0.21 (0.03, 1.44) | 0.112 |
|  | Bipolar disorder | 277 (61) | | **1.77 (1.13, 2.75)** | 0.012 | 238 (51) | 1.60 (1.02, 2.50) | 0.042 | 94 (22) | 1.40 (0.64, 3.03) | 0.397 |
|  | Antipsychotics | 766 (163) | | 1.15 (0.88, 1.50) | 0.301 | 601 (128) | 1.12 (0.84, 1.50) | 0.441 | 386 (79) | 0.91 (0.63, 1.32) | 0.624 |
|  | Lithium | 145 (30) | | 1.64 (0.81, 3.31) | 0.171 | 132 (27) | 1.13 (0.56, 2.28) | 0.732 | 34 (7) | 2.61 (0.70, 9.70) | 0.151 |
|  | Antiepileptics | 651 (145) | | 1.16 (0.87, 1.56) | 0.313 | 504 (110) | 1.11 (0.81, 1.54) | 0.510 | 271 (57) | 1.00 (0.64, 1.55) | 0.988 |
| Neurodevelopmental conditions | Attention-Deficit/Hyperactivity Disorder | 693 (155) | | 1.02 (0.79, 1.30) | 0.905 | 617 (136) | 1.00 (0.76, 1.33) | 0.978 | 348 (71) | **1.81 (1.29, 2.55)** | 0.001 |
|  | Tic disorder | 42 (11) | | 4.94 (0.71, 34.57) | 0.107 | 40 (10) | 2.55 (0.30, 21.42) | 0.388 | 30 (7) | 1.64 (0.26, 10.56) | 0.601 |
|  | Autism spectrum disorder | 269 (60) | | 1.00 (0.61, 1.64) | 0.992 | 185 (42) | 0.85 (0.46, 1.54) | 0.582 | 112 (24) | 1.01 (0.38, 2.65) | 0.991 |
|  | Intellectual disability | 118 (28) | | 1.41 (0.67, 3.01) | 0.366 | 91 (21) | 2.08 (1.03, 4.19) | 0.042 | 53 (13) | 0.40 (0.13, 1.27) | 0.121 |
|  | Learning disorders | 36 (8) | | 0.27 (0.07, 1.00) | 0.049 | 33 (7) | 0.71 (0.13, 3.75) | 0.682 | 12 (2) | NA | NA |
|  | Stimulants | 789 (174) | | 1.12 (0.89, 1.41) | 0.341 | 688 (149) | 1.08 (0.83, 1.40) | 0.572 | 409 (84) | **1.84 (1.31, 2.59)** | <0.001 |
| Internalizing conditions | Anxiety | 1178 (260) | | 1.19 (0.98, 1.44) | 0.074 | 932 (206) | 1.17 (0.94, 1.47) | 0.166 | 552 (118) | 1.37 (1.06, 1.76) | 0.016 |
|  | Depression | 1162 (256) | | 1.07 (0.89, 1.29) | 0.467 | 922 (203) | 1.12 (0.91, 1.38) | 0.293 | 530 (112) | 0.96 (0.74, 1.25) | 0.760 |
|  | Post-traumatic stress disorder | 819 (171) | | 1.17 (0.92, 1.48) | 0.209 | 654 (136) | 1.20 (0.92, 1.58) | 0.181 | 391 (78) | **2.10 (1.46, 3.03)** | <0.001 |
|  | Obsessive-compulsive disorder | 205 (42) | | 0.89 (0.53, 1.49) | 0.656 | 209 (41) | 0.79 (0.50, 1.26) | 0.330 | 57 (13) | 0.31 (0.12, 0.79) | 0.014 |
|  | Eating disorders | 233 (51) | | 0.83 (0.52, 1.34) | 0.446 | 203 (44) | 1.39 (0.82, 2.37) | 0.224 | 112 (22) | 0.75 (0.31, 1.80) | 0.521 |
|  | Anxiolytics | 2434 (565) | | **1.22 (1.08, 1.37)** | 0.001 | 1782 (410) | **1.23 (1.07, 1.42)** | 0.003 | 1040 (233) | 0.86 (0.72, 1.03) | 0.106 |
|  | Antidepressants | 2595 (593) | | **1.25 (1.12, 1.40)** | <0.001 | 1903 (437) | 1.15 (1.01, 1.31) | 0.040 | 1126 (250) | 0.95 (0.80, 1.12) | 0.515 |
| Substance use disorders | Alcohol-related disorders | 686 (144) | | 1.27 (0.99, 1.62) | 0.063 | 450 (96) | 1.24 (0.90, 1.71) | 0.179 | 411 (82) | 1.05 (0.77, 1.44) | 0.741 |
|  | Drug-related disorders | 453 (98) | | 1.27 (0.88, 1.85) | 0.206 | 346 (76) | 1.36 (0.90, 2.05) | 0.142 | 275 (53) | 1.25 (0.81, 1.93) | 0.321 |
|  | Anti-alcohol medications | 283 (55) | | 1.49 (0.91, 2.44) | 0.111 | 178 (33) | 0.81 (0.44, 1.51) | 0.511 | 182 (33) | 1.03 (0.54, 1.96) | 0.926 |
|  | Anti-opioid medications | 36 (7) | | 0.55 (0.10, 3.15) | 0.505 | 40 (7) | 1.29 (0.27, 6.23) | 0.752 | 7 (1) | NA | NA |
| Externalizing behaviors | Oppositional defiant disorder | 46 (11) | | 1.67 (0.66, 4.25) | 0.283 | 37 (9) | 1.08 (0.35, 3.37) | 0.893 | 33 (8) | 3.57 (1.01, 12.59) | 0.048 |
|  | Violent crimes | 551 (120) | | 1.02 (0.75, 1.39) | 0.884 | 361 (78) | 0.87 (0.59, 1.28) | 0.481 | 383 (81) | 1.22 (0.89, 1.67) | 0.224 |

^a^ Sample size of the cousins (i.e., children of monozygotic twins) who were discordant for both exposure and outcome. The sample size of the cousin cluster was in parentheses. Hazard ratio was not estimated for those sample sizes of cousin cluster less than 5. We performed stratified Cox regression to estimate the within-twin-family effects. Adjusted covariates include the highest parental educational level, any partner psychiatric conditions, maternal and paternal age of childbirth, offspring birth year, and sex. Estimators shown in bold meet the criteria for Benjamini-Hochberg False Discovery Rate statistical significance (N = 24*3).

**References**

1. Ludvigsson JF, Almqvist C, Bonamy AK, et al. Registers of the Swedish total population and their use in medical research. Eur J Epidemiol 2016;31(2):125–36.

2. Ekbom A. The Swedish Multi-generation Register. Methods in Molecular Biology: Humana Press; 2011:215–220.

3. Socialstyrelsen. The National Patient Register. (<https://www.socialstyrelsen.se/en/statistics-and-data/registers/national-patient-register/>).

4. Ludvigsson JF, Andersson E, Ekbom A, et al. External review and validation of the Swedish national inpatient register. BMC Public Health 2011;11(1):450.

5. Wettermark B, Hammar N, Fored CM, et al. The new Swedish Prescribed Drug Register--opportunities for pharmacoepidemiological research and experience from the first six months. Pharmacoepidemiol Drug Saf 2007;16(7):726–35.

6. Frisell T, Lichtenstein P, Langstrom N. Violent crime runs in families: a total population study of 12.5 million individuals. Psychol Med 2011;41(1):97–105.

7. Brooke HL, Talback M, Hornblad J, et al. The Swedish cause of death register. Eur J Epidemiol 2017;32(9):765–773.

8. Ludvigsson JF, Svedberg P, Olen O, Bruze G, Neovius M. The longitudinal integrated database for health insurance and labour market studies (LISA) and its use in medical research. Eur J Epidemiol 2019;34(4):423–437.

9. Lichtenstein P, De Faire U, Floderus B, Svartengren M, Svedberg P, Pedersen NL. The Swedish Twin Registry: a unique resource for clinical, epidemiological and genetic studies. J Intern Med 2002;252(3):184–205.
