# Supplementary material for: Association between parental psychiatric conditions and offspring psychiatric, behavioral, and psychosocial outcomes: A Swedish population-based children-of-monozygotic twins study
Source: PLoS Med. 2025 Oct 21;22(10):e1004784. doi: 10.1371/journal.pmed.1004784 (PMC12571287; doi:10.1371/journal.pmed.1004784)
Supplement: S2 Appendix — (PDF) [file pmed.1004784.s003.pdf]

Psych4: Project Proposal

Submit project proposals & the excel sheet for paper proposal to isabell.brikell@ki.se after reviewing the publication policy, the Psych4 project plan and ethical approval. Projects will be evaluated at the monthly steering committee meeting.

Project title: Intergenerational Transmission between Parental Psychiatric Conditions and Offspring Psychiatric, Behavioral, and Psychosocial Outcomes: A Swedish Population-Based Children-of-Monozygotic Twins Study

PI/responsible researcher: Erik Pettersson

Grad student: Mengping Zhou

Key collaborator from the Psych4 steering group: Ralf Kuja-Halkola, Zheng Chang, Isabell Brikell, Paul Lichtenstein & Henrik Larsson

Relevance to Psych4 ethical approval: Existing studies found vast associations between parental psychiatric conditions and offspring outcomes. However, these were observational studies that cannot inform on whether treating the parental conditions would alleviate the offspring outcomes (i.e., whether the associations are causal). Investigating the causal pathway through parental psychiatric conditions to offspring adverse outcomes aligns with the goals stated in the Psych4 project plan ("to understand how genes and environments influence the intergenerational transmission").

Abstract (half page including below sections):

Brief background: Mental health problems often run in families, with studies showing transdiagnostic associations across generations. Nevertheless, if these associations were attributable to unmeasured familial (either environmental or genetic) factors that influence both generations, then treating the parental conditions would not break the intergenerational transmission.

Research questions: Whether the associations between parental psychiatric conditions and offspring psychiatric, behavioral, and psychosocial outcomes remain after controlling for unmeasured familial factors shared by offspring of monozygotic (MZ) twin parents (i.e., cousins).

Population: Individuals born to MZ twin parents in Sweden between 1970 and 2000.

Intervention / Exposure: The exposures were whether parents were diagnosed with any internalizing or externalizing condition. We defined internalizing conditions based on whether parents had any lifetime diagnoses of anxiety, depression, post-traumatic stress disorder (PTSD), obsessive-compulsive disorder (OCD), or eating disorders. We defined externalizing conditions based on whether parents had any lifetime diagnoses of substance use disorders (i.e., alcohol-related disorders, drug-related disorders), oppositional defiant disorder (ODD), or a court conviction of a violent crime.

Comparison / Control: Offspring born to a MZ twin parents without the diagnosis

Outcome: Psychiatric, behavioral, and psychosocial outcomes in offspring included 15 psychiatric diagnoses, 8 prescriptions of medications, court convictions of violent crimes, school performance, long-term unemployment, and suicide.

Time period/follow-up: We followed individuals from their date of birth to the date of the outcome, emigration from Sweden, death, or December 31, 2020, whichever occurred first.

Planned analyses: We will first compare offspring exposed to parental psychiatric conditions to offspring not exposed, generating between-family estimates. Second, we will compare individuals to their cousins born to MZ twin parents (i.e., where one twin parent had a psychiatric condition, and the other twin parent did not), generating within-twin-family estimates. To assess the between-family effects, we will perform Cox regression for time-to-event outcomes and logistic regression for binary outcomes. To estimate the within-twin-family effects (i.e., fixed-effects or cousin comparison model), we will perform stratified Cox regression or conditional logistic regression, depending on the outcome.

Required data/tables & variables: see list of ICD codes

Financing agreement obtained (Y/N): (if not contact Henrik Larsson): Y

Contract needed (Y/N): No

Planned papers: Please fill in the Psych4_paper_proposal_template for each planned paper. For larger projects/grants, additional papers may be added at a later time point.

Required registers:

Total Population Register

Multi-Generation Register

National Patient Register

Prescribed Drug Register

National Crime Register

National School Register

Cause of Death Register

Longitudinal Integration Database for Health Insurance and Market Studies

Swedish Twin Register

Exposure/outcome	ICD 08 (1969-1986)	ICD 09 (1987-1996)	ICD 10 (1997-)	ATC code	Minimum age
Schizophrenia	295 (except 295.5)	295 (except 295F)	F20, F25		15
Bipolar disorder	296.1, 296.3, 296.8, 296.9	296A, 296C, 296D, 296E, 296W, 296X	F30, F31		15
Depression	300.4	296B, 311, 300E	F32, F33		10
Anxiety	300 (except 300.3, 300.4)	300, (except 300D, 300E)	F40, F41		10

Exposure/outcome	ICD 08 (1969-1986)	ICD 09 (1987-1996)	ICD 10 (1997-)	ATC code	Minimum age
Obsessive-compulsive disorder	300.3	300D	F42		5
Post-traumatic stress disorder	307.99	308, 309	F43		2
Alcohol-related disorders	291, 303	291, 303, 305A	F10 (except F10.5)		12
Drug-related disorders	304	292, 304, 305X	F11-F19 (except F17, F1x.5)		12
Attention-Deficit/Hyperactivity Disorder	-	314	F90		3
Autism spectrum disorder	-	299A	F84.0, F84.1, F84.5		2
Tic disorder	306.2	307C	F95		3
Learning disorders	-	315A, 315B	F81		3
Intellectual disability	311-315	317-319	F70-F79		2
Oppositional defiant disorder	-	312X	F91		3
Eating disorders	784.00	307B, 307F	F50		10
Suicide (Suicide attempts and death by suicide)	E950-959 E980-989	E950-959 E980-989	X60-X84, Y10-Y34		10
Anti-alcohol medication				N07BB	12
Antidepressants				N06A	10
Antiepileptics				N03A	3
Anti-opioid medication				N07BC	12
Antipsychotics				N05AA-N05AL, N05AX	15

Exposure/outcome	ICD 08 (1969-1986)	ICD 09 (1987-1996)	ICD 10 (1997-)	ATC code	Minimum age
Anxiolytics				N05B	10
Lithium				N05AN	10
Stimulants				N06B	3
Violent crimes	homicide (Ch 3, §1-3); assault (Ch 3, §5-6); robbery (Ch 8, §5-6); threats and violence against an officer (Ch 17, §1-2); gross violation of a person's/woman's integrity (Ch 4, §4a); unlawful coercion (Ch 4, §4); unlawful threats (Ch 4, §5); kidnapping (Ch 4, §1); illegal confinement (Ch 4, §2); arson (Ch 13, §1-2); intimidation (Ch 4, §7); sexual offence (excluding prostitution and the buying of sexual services but including child pornography) (Ch 6 §1-10, §10A, §12)				15
Poor school performance	Defined as ranking in the lowest quintile on the junior high school grade point average, or attaining only a compulsory education level				10
Long-term unemployment	Defined as at least one consecutive year unemployment				18
