# Supplementary material for: Association between parental psychiatric conditions and offspring psychiatric, behavioral, and psychosocial outcomes: A Swedish population-based children-of-monozygotic twins study
Source: PLoS Med. 2025 Oct 21;22(10):e1004784. doi: 10.1371/journal.pmed.1004784 (PMC12571287; doi:10.1371/journal.pmed.1004784)
Supplement: Attachment — Submitted filename: Response letter.docx [file pmed.1004784.s006.docx]

Dear editors and reviewers,

Thank you for your insightful comments and suggestions. We have carefully considered all the comments and made revisions and adjustments accordingly in our manuscript.

As a general comment, because several reviewers raised concerns about the lack of statistical power and capped follow-up time, we have reanalyzed all models using an updated registry linkage with extended follow-up through December 31, 2020 - the most recent data available (previously end of follow-up was December 31, 2013). This extension yielded increases in effective sample sizes for cousin clusters discordant for both exposures and outcomes. Subsequently, we observed that 7 of the 27 associations remained statistically significant in the within-family models after multiple testing correction (in the previous linkage with follow-up until only 2013, the corresponding number of significant associations was 0 out of 18; the total number of associations increased to 27 because we now include any parental psychiatric disorder as an exposure, based on reviewer feedback). Specifically, across the main analysis and several sensitivity analyses, statistically significant associations remained between any parental psychiatric condition and any offspring psychiatric condition. We have now revised our conclusions accordingly throughout the manuscript. Furthermore, in response to reviewer feedback, we also acknowledge that the statistical power for some specific clustered outcomes is limited even with the updated linkage.

On page 3 in the revised Abstract section, we now write:

***Results:*** *……Although these associations attenuated when comparing differentially exposed cousins whose parents were MZ twins (20 out of 27 associations were no longer statistically significant within cousin pairs), associations between broad spectra remained statistically significant. Specifically, across* *the main analysis and several sensitivity analyses, significant within-cousin pair associations remained between any parental psychiatric condition and any offspring psychiatric condition (HR = 1.28, 95% CI: 1.13, 1.44), between parental internalizing conditions and any offspring psychiatric condition (HR = 1.26, 95% CI: 1.09, 1.45), and between parental externalizing conditions and any offspring psychiatric condition (HR = 1.27, 95% CI: 1.08, 1.51).*

***Conclusions:*** *Although the intergenerational transmission between parental psychiatric conditions and offspring psychiatric, behavioral, and psychosocial outcomes appeared partially attributable to unmeasured familial (environmental or genetic) factors that influenced both generations, there was also evidence of either non-shared confounders or direct causal effects. If the latter, then treating parental psychiatric conditions would reduce the risk of psychiatric vulnerability in offspring.*

On page 21 in the revised Limitation section, we now write:

*Seventh, although our study represents the largest children-of-identical-twins sample to date, statistical power remained limited for specific clustered outcomes, preventing us from drawing strong conclusions regarding these outcomes.*

**Please find our responses below to all comments and descriptions of the revisions we made in our updated manuscript.**

**Reviewer #1 (statistics):** 
This is an interesting study that examined the intergenerational transmission between parental psychiatric conditions and their offspring's outcomes. It could have strong implication on how the transmission should be tackled, and the use of a children-of-monozygotic twins design is excellent in handling how familial / genetic factors being controlled.  
The study is well conducted and the reporting is with sufficient details. The statistical methods were appropriate. The findings are important and interesting. I have several minor comments: 
1.1 I'm a bit reserved about the claim that the non-significant findings means no associations, i.e. the claim 'fully attributable to unmeasured familial and genetic factors' in the conclusion. The estimated 95% CIs are quite wide (0.45 (95% CI: 0.20, 1.02) to 1.57 (95% CI: 0.83, 2.96)) and still compatible with strong associations.

**Response:** We appreciate this important observation and agree that non-significant findings should not necessarily be interpreted as evidence of no association, particularly given the wide confidence intervals. As detailed in the General comment above, we have a) revised our conclusions in light of the updated linkage and b) interpreted the null associations more cautiously.

1.2 If indeed authors want to provide stronger support to this claim, a post-hoc power analysis may be able to show detectable effect size from this study.

**Response:** As detailed in the General comment above, following a reanalysis of all models in an updated registry linkage with extended follow-up through December 31, 2020, several within-cousin pair associations remained significant. Therefore, we think a post-hoc power analysis might be redundant.

1.3 The end of follow-up was quite dated - over 10 years ago. Would there be any notable diagnostic differences that makes the findings less applicable to today? A short discussion may be useful.

**Response:** As detailed in the General comment above, we have now extended our follow-up period through December 31, 2020. This extended follow-up enhances the contemporary relevance and applicability of our findings.

**Reviewer #2:** 
The present study examines intergenerational transmission of mental disorders using children of twins approach in Swedish registry data. Results demonstrate that although parental mental disorders were related with increased risk of offspring mental disorders, the association were diluted in the within-twin-family analyses. Although the present study approach is novel and the results are very interesting, there are number of issues that require further clarification.
2.1 Although study is based on large-scale register, the statistical power (based on table 2) seems rather limited in many of the analyses. Most of the data is currently available until to the end of 2013. Would be possible to update the data up to 2023, for example, to increase statistical power?

**Response:** As detailed in the General comment above, we have reanalyzed all models in an updated registry linkage with extended follow-up through December 31, 2020, which increased our effective sample sizes and statistical power.

2.2 Using life-time diagnosis as the main exposure seems like not the best possible solution, and rational for this is currently missing. The first sensitivity analyses partially address this issue, but ideally parental mental disorders would be diagnosed before the follow-up for offspring mental disorders begins to avoid reverse causality.

**Response:** We used lifetime parental psychiatric diagnoses based on several key considerations. First, well-documented delays exist between the onset of psychiatric symptoms and the assignment of a clinical diagnosis, particularly for more severe disorders such as bipolar disorder, where studies consistently report diagnostic lags of 5-10 years [1, 2]. Restricting analyses to parents whose diagnoses occurred before childbirth would therefore exclude a substantial portion of parents with psychiatric liability during critical offspring developmental periods, significantly reducing statistical power and potentially biasing the true intergenerational transmission risk. Second, psychiatric diagnoses likely partially reflect an underlying trait-like liability, as indicated by their moderate to high heritability estimates and substantial stability over time [3, 4]. While formal diagnoses might be assigned post-symptom onset, this latent liability might still influence the offspring's developmental environment. Finally, in a prior (observational) study we conducted on intergenerational transmission patterns, the results remained the same regardless of whether we treated the parental diagnoses as time-constant versus time-varying exposures, suggesting that the liability toward psychiatric conditions captures a sizeable portion of the risk [5]. We have explicitly acknowledged using lifetime parental diagnoses as a limitation.

On page 21 in the revised Limitation section, we now write:

*Ninth, our use of lifetime parental diagnoses did not ensure temporal precedence, as some conditions may have been diagnosed before the birth or after the outcome of their child. While our sensitivity analyses restricting parental diagnoses occurring before the offspring's adolescence (before age 18) and to the post-childbirth period demonstrated robust effects, future studies could employ stricter temporal definitions for exposure.*

2.3 The interpretation of the findings warrants further discussion, as the between-family and within-family models target different causal estimands (the first estimates family-level causal effect and the second family level causal export among exposure-discordant cousins). Thus, the findings from these two models are not directly comparable without additional assumptions.

**Response:** Although between- and within-cluster models target different estimands, both the between-family and within-twin-family analyses were conducted in the same sample of offspring born to monozygotic twin parents. We agree with the reviewer that the between-family estimates the global population-level effect by comparing all offspring exposed to parental psychiatric conditions with all unexposed offspring, capturing the total association, including both direct causal effects, confounding from shared familial factors such as genetics, environment, and socioeconomic status, and confounding from non-shared factors. In contrast, the within-twin-family estimates a conditional effect within family strata by comparing exposed offspring to their unexposed cousins within the same twin family, thereby controlling for all familial factors shared by the monozygotic twin parents. Rather than targeting incomparable causal estimands, these models form part of an established decomposition strategy in family-based causal inference [6, 7].

On page 11 in the revised Method section, we now write:

*The between-family analyses compare all offspring exposed to parental psychiatric conditions against all unexposed offspring, thereby capturing (potential) direct causal effects, confounding from shared familial factors (e.g., genetics, environment, and socioeconomic status), and confounding from non-shared factors. In contrast, the within-twin-family estimates are conditional on family strata, thereby controlling for confounding from shared familial factors.*

2.4 Given the high lifetime incidence of mental disorders (up to 80% in Kessing et al. JAMA Psychiatry 2023), the prevalence of parental mental disorders in the study cohorts seems rather low. What could explain this and how does it influence the present findings?

**Response:** Yes, while Kessing et al. reported a cumulative incidence of 29% for hospital-contact diagnoses and 82.6% when including psychotropic prescriptions [8], our study identified a somewhat lower estimate of any psychiatric diagnosis in 20.7% of the offspring. This discrepancy arises from key methodological differences: our ascertainment relied exclusively on psychiatrist-assigned diagnoses (and not psychotropic prescriptions). That is, we only include help-seeking individuals who received a psychiatric diagnosis after following up on a referral from a primary care physician, or after going directly to an emergency psychiatric in- or out-patient unit (or who were forcibly admitted, though this captures only a sliver of the population). Furthermore, the offspring follow-up ended at age 51 in our study, rather than age 100 as in Kessing's study, potentially missing later-onset conditions.

This potential under-ascertainment might affect our findings in several ways. First, we are probably capturing cases identified in specialist care, so the associations likely generalize primarily to clinically significant conditions rather than milder manifestations. Second, reduced prevalences constrain statistical power by limiting discordant cousin clusters. Third, surveillance bias may occur if offspring of affected parents seek care more readily, leading to differential outcome misclassification, the effect of which is difficult to predict (i.e., it could lead to either under- or overestimated associations). Exposure misclassification, on the other hand, tends to be nondifferential and leads to underestimated associations [9]. On the whole, the net effect of surveillance bias on intergenerational transmission estimates is therefore somewhat difficult to predict.

On page 20 in the revised Limitation section, we now write:

*Fourth,* *we included only clinical diagnoses assigned in specialist care (i.e., we lacked information on individuals treated exclusively in primary care or those who did not seek treatment), so the associations are likely generalizable to cases identified in specialist care only.*

2.5 In addition to examining parental externalizing and internalizing disorders, I would like to see an analysis where any parental mental disorders would be used as an exposure.

**Response:** We now include an analysis using any parental psychiatric disorder as an exposure variable. The results demonstrate that any parental psychiatric condition was significantly associated with any offspring psychiatric condition, and with offspring internalizing conditions, both between- and within-cousin clusters and across various sensitivity specifications.

2.6 Discussion related to the validity of the mental disorders diagnoses is currently missing.

**Response:** We have now added a discussion of diagnostic validity to our manuscript.

On page 21 in the revised Limitation section, we now write:

*Sixth, although a study found a high positive predictive value (PPV) of 84% for psychiatric diagnoses [10], the accuracy varies across conditions. Specifically, the corresponding PPV was 81% for bipolar disorder [11], 72% for obsessive-compulsive disorder [12], and 92% for tic disorders [12]. This variation in diagnostic accuracy may introduce different misclassifications across psychiatric conditions, which we assume is nondifferential with respect to exposure and outcome,* *a scenario that would typically underestimate true associations [9].*

**Reviewer #3:** 
Zhou and colleagues investigate the intergenerational transmission of psychiatric conditions using high-quality Swedish national registry data and a sophisticated children-of-MZ-twins (CoMZ) design. Strengths of this study include the use of clinician-assigned psychiatric diagnoses, a broad range of offspring psychiatric and psychosocial outcomes, and thoughtful sensitivity analyses. The manuscript is well-written, the analyses are rigorous, and the Discussion is nuanced and appropriately contextualized. Below, I provide specific suggestions to further strengthen the manuscript.
3.1 The claim that intergenerational transmission is "fully attributable" to familial factors may be overstated. While the attenuation of associations in the within-twin-family models indeed reflect familial factors, the substantial reduction in effective sample size (e.g., some outcomes had fewer than 100 discordant cousin clusters) could limit the power to detect small but potentially meaningful environmental effects. I would suggest that authors interpret these non-significant findings more cautiously. Additionally, the Discussion would benefit from further consideration of the limited statistical power inherent in the CoMZ design, the possibility of residual confounding, and the fact that some associations (e.g., between parental internalizing conditions and offspring school performance or unemployment) became statistically significant in the age-restricted sensitivity analysis.

**Response:** As detailed in the General comment above, we have a) reanalyzed all models in an updated registry linkage with extended follow-up time that boosted statistical power, and b) interpreted null findings more cautiously. We now discuss the possibility of residual confounding.

On page 20 in the revised Limitation section, we now write:

*First, importantly, despite controlling for unmeasured familial factors shared by cousins, the COT designs cannot isolate unmeasured confounding not shared by cousins. Although we controlled for potential observed non-shared confounders, such as psychiatric diagnoses of the twin parents' partners, unobserved residual confounding could remain.*

3.2 While transdiagnostic approaches are valuable, the rationale for grouping specific psychiatric conditions into internalizing and externalizing domains should be more clearly explained. For example, the inclusion of PTSD, OCD, and eating disorders under internalizing conditions warrants justification. Additionally, while parental psychiatric conditions were grouped into two broad domains (internalizing vs. externalizing), offspring outcomes were analyzed across nine distinct clusters. Although the authors mention this was done to increase power, a clearer theoretical or methodological rationale for the asymmetry between exposure and outcome domains would strengthen the justification.

**Response:** We grouped psychiatric conditions into internalizing and externalizing domains based on established transdiagnostic frameworks [13, 14]. To empirically validate this grouping, we have conducted an exploratory factor analysis (EFA) of the parental conditions. As shown in Table 1 below, a two-factor EFA revealed a clear latent structure: depression (loading = 0.81), anxiety (loading = 0.79), PTSD (loading = 0.63), OCD (loading = 0.69), and eating disorders (loading = 0.69) were associated with the first factor, supporting their inclusion within the internalizing domain. Conversely, oppositional defiant disorder (loading = 0.50), violent crime (loading = 0.73), drug-related disorders (loading = 0.68), and alcohol-related disorders (loading = 0.83) were associated with the second factor, confirming their classification as externalizing conditions. This empirical validation supports our conceptually-based grouping of conditions into the internalizing-externalizing spectra, and seems to justify grouping PTSD, OCD, and eating disorders within the internalizing domain.

**Table 1. Factor loadings of two-factor EFA based on 9 psychiatric conditions**

| **Diagnosis** | **Factor 1** | **Factor 2** |
| --- | --- | --- |
| Depression | **0.81** | 0.36 |
| Anxiety | **0.79** | 0.39 |
| PTSD | **0.63** | 0.39 |
| OCD | **0.69** | 0.07 |
| Eating disorder | **0.69** | 0.02 |
| Oppositional defiant disorder | 0.27 | **0.50** |
| Violent crime | -0.06 | **0.73** |
| Drug related disorder | 0.25 | **0.68** |
| Alcohol related disorder | 0.35 | **0.83** |

*Note.* Loadings greater than 0.40 are bolded for visual clarity.

Regarding the asymmetry between exposure (parental conditions) and outcome (offspring clusters) domains, the decision was driven by maximizing statistical power while capturing the breadth of potential intergenerational effects. We have now explained the rationale in the manuscript.

On page 9 in the revised Method section, we now write:

*We did not include parental psychotic and neurodevelopmental disorders as exposures for three reasons. First, these two exposures had limited statistical power due to low prevalences. Second, as discussed in the Introduction, evidence suggests that the intergenerational transmission of psychotic disorders appears almost entirely attributable to familial factors, making their inclusion less meaningful. Third, the primary neurodevelopmental diagnoses were not included when the inpatient registry began in 1973, which would lead to under-ascertainment of neurodevelopmental diagnoses among parents born before 1960 (corresponding to 66% of our sample), given their characteristic of early-life onset.*

3.3 Although Tables 1 and 2 provide detailed sample size information, the effective sample sizes used in the between-family and within-twin-family analyses are not explicitly stated in the Results section. Given that the interpretation of null findings depends on statistical power, I recommend briefly reiterating the analytic sample sizes, particularly the number of discordant cousin pairs contributing to the within-family models (e.g., range of N across outcomes), either at the beginning of the Results or within each relevant subsection.

**Response:** We have now added this description to the manuscript.

On page 13 in the revised Results section, we now write:

*The sample sizes of* *both exposure- and outcome-discordant cousins (cousin clusters) ranged from 577 (127) for externalizing behaviors to 2,801 (650) for internalizing conditions when examining the association with any parental psychiatric condition; from 390 (85) for externalizing behaviors to 1,996 (462) for internalizing conditions when examining associations with parental internalizing conditions; and from 321 (63) for suicide to 1,176 (265) for internalizing conditions when examining associations with parental externalizing conditions.*

3.4. The finding that parental internalizing conditions were significantly associated with poor school performance and long-term unemployment in the sensitivity analysis restricting cousin age difference (≤8 years) is important. I suggest more explicitly exploring potential explanations in the Discussion.

**Response:** As detailed in the General comment above, we have updated our analyses with extended follow-up through December 31, 2020. In the updated results, we observed that parental internalizing conditions were significantly associated with poor school performance, and that parental internalizing conditions were significantly associated with both poor school performance and long-term unemployment in the sensitivity analysis when restricting cousin age difference (≤8 years). Although we agree that this is an important finding, we are somewhat concerned that this association did not emerge as statistically significant across all the sensitivity analyses. In addition, as we discussed in the manuscript, a Norwegian register-based adoption study observed a null association between adoptive parents’ internalizing disorders and school performance among the adoptees [15]. Therefore, we recommended that further studies using genetically informative designs or other natural experiments are warranted to explore whether our significant within-twin-family estimate replicates.

On page 21 in the revised Limitations section, we now write:

*Tenth, although associations remained significant for certain specific outcomes in the within-family model (e.g., parental internalizing conditions were associated with offspring poor school performance), these associations were not statistically significant across all the sensitivity analyses. Therefore, it is important to explore if this association replicates in other samples or with other quasi-experimental designs.*

3.5. While partner psychiatric diagnosis is included as a covariate, it may not fully account for intergenerational effects arising from assortative mating or genetic nurture. These mechanisms could contribute to residual confounding in both between- and within-family models. A brief discussion of how these complexities might influence the current findings would be informative and help contextualize the strengths and limitations of the CoMZ design.

**Response:** While assortative mating is well-established between families [16], its impact in within-cousin comparison models depends on its underlying drivers. Assortative mating driven by genetic factors or shared environment can be controlled by the model. In contrast, assortative mating arising from unique environmental factors or random choice may evade control, potentially leading to overestimated associations. Supporting the genetic drive hypothesis, polygenic risk score studies demonstrated that partners exhibited significant genome-wide similarity for mental health-related traits [17, 18]. Supporting the unique environmental drivers, one study of twin mate selection found that while individual twins showed modest personality similarity with their own spouses (*r* = 0.13), their spouses exhibited virtually no personality similarity with each other (*r* = 0) [19]. Given that personality traits appear to capture the lower end of psychiatric spectra [20], this suggests that specific partner choice (with respect to the variance shared by personality traits and psychiatric conditions) might be random among genetically identical individuals, implying assortative mating is driven by unique environmental factors. Critically, partner choice might function not as a confounder (as typically assumed in assortative mating scenarios) but as a mediator. If so, controlling for partner characteristics could lead to over-correction and underestimated associations.

Regarding genetic nurture - where parental genotypes influence offspring through environmental pathways - this should not confound our within-MZ twin comparisons. MZ twins share identical genetics, meaning any genetic nurture effects from the twin parents ought to be equivalent within pairs [21].

On page 21 in the revised Limitation section, we now write:

*Eighth, although we controlled for partner psychiatric diagnosis, residual confounding from assortative mating might persist in both the between- and within-family models, which could* *potentially lead to overestimated associations. However, partner choice might function not as a confounder (as typically assumed in assortative mating scenarios) but as a mediator. If so, controlling for partner characteristics could constitute over-correction and lead to underestimated associations.*

3.6. Recent family-based genetic studies increasingly use polygenic risk scores (PRS) from both parents and offspring to investigate intergenerational psychiatric risk. I recommend briefly situating the current findings within this growing body of literature and explaining how the CoMZ design complements these genome-based approaches.

**Response:** Family-based polygenic risk scores (PRS) studies can identify the quasi-causal effect of genetics and quantify genetic nurture effects. Recent PRS studies from parents and/or offspring consistently demonstrate that genetic nurture appears quite small for psychiatric conditions, implying that direct genetic transmission constitutes the primary mechanism in intergenerational transmission of mental health problems [22-27].

Nevertheless, PRS studies are typically limited in that they often only capture a fraction of the total genetic liability, and thereby explain limited phenotypic variance for most psychiatric traits [21]. In contrast, the CoMZ design controls for all shared genetic factors between the twin parents, thereby estimating the direct phenotypic effects of parental mental health conditions on offspring outcomes. Thus, although the CoMZ design cannot identify genetic nurture effects like PRS studies, it might have greater real-world applications.

Findings from both approaches underscore that genetic factors significantly inflate family-based estimates of intergenerational psychiatric risk transmission, highlighting the importance of controlling for unmeasured genetic factors when attempting to investigate the causal effect of parental psychiatric conditions on offspring outcomes.

On page 17 in the revised Discussion section, we now write:

*Although our study did not decompose the familial factors into that which can be attributed to genetic versus* *shared environmental factors, we speculate that genetic factors likely feature centrally in the intergenerational transmission because the unmeasured environmental factors shared by cousins that are adjusted for in the COT design are often relatively small [28]. Indeed, a recent systematic review that included studies that decomposed familial factors into genetic and shared environmental subparts also concluded that genetic transmission plays an important role [21]. In addition, recent family-based polygenic risk scores studies based on parents and/or offspring consistently demonstrate that direct genetic transmission constitutes the primary mechanism in intergenerational transmission of mental health problems [22-27].*

Other minor comments:
3.7 Please add a clear explanation of the distinction between between-family and within-twin-family analyses.

**Response:** As detailed in our response to comment 2.3 above, the between-family estimates the global population-level effect by comparing all offspring exposed to parental psychiatric conditions with all unexposed offspring, capturing the total association, including both direct causal effects, confounding from shared familial factors such as genetics, environment, and socioeconomic status, and confounding from non-shared factors. In contrast, the within-twin-family estimates a conditional effect within family strata by comparing exposed offspring to their unexposed cousins within the same twin family, thereby controlling for all familial factors shared by the monozygotic twin parents.

On page 12 in the revised Method section, we now write:

*The between-family analyses compare all offspring exposed to parental psychiatric conditions against all unexposed offspring, thereby capturing (potential) direct causal effects, confounding from shared familial factors (e.g., genetics, environment, and socioeconomic status), and confounding from non-shared factors. In contrast, the within-twin-family estimates are conditional on family strata, thereby controlling for confounding from shared familial factors.*

3.8 Please clarify how missing data (e.g., covariates, psychiatric outcomes) were handled, for instead complete case analysis, imputation, or otherwise.

**Response:** We employed complete case analysis, excluding individuals with missing values on any covariates. However, given that only 0.6% of our sample had missing values on covariates, we consider any potential bias as negligible. Regarding psychiatric outcomes, we included only clinical diagnoses assigned in specialist care using the National Patient Register, which by design has no missingness. Nevertheless, as detailed in our response to comment 2.4 above, this likely captures cases identified in specialist care, and misses individuals treated exclusively in primary care or those who did not seek treatment.

On page 20 in the revised Limitation section, we write:

*Fourth, we included only clinical diagnoses assigned in specialist care (i.e., we lacked information on individuals treated exclusively in primary care or those who did not seek treatment), so the associations are likely generalizable to cases identified in specialist care only.*

3.9 Justify the rationale for selecting an 8-year cousin age gap threshold in the sensitivity analysis. Citing a relevant reference or providing examples (e.g., changes in diagnostic practices or healthcare access) would improve interpretability.
**Response:** Although not reported in the manuscript, we conducted sensitivity analyses using different cousin age gap thresholds (i.e., 6, 8, and 10 years). All three gap thresholds yielded similar results. We therefore selected to report only the 8-year threshold because a) the results remained the same across the three specifications, b) it provides sufficient temporal separation to minimize potential period effects (e.g., changes in diagnostic practices, healthcare access) while retaining adequate sample sizes for statistical power, c) it aligns with our previous sibling comaprison study [29].

On page 12 in the revised Method section, we now write:

*We selected an 8-year cousin age gap because it provides sufficient temporal separation to minimize potential cohort effects (e.g., changes in diagnostic practices, healthcare access) while retaining adequate sample sizes for statistical power and aligns with our previous sibling comparison study [29].*

3.10 If the sample permits, consider testing or discussing potential sex differences in intergenerational transmission patterns.

**Response:** Following the reviewer’s suggestion, we conducted exploratory stratified analyses by parent sex (mothers vs. fathers) and offspring sex (female vs. male offspring) to assess potential sex-specific patterns. As shown in Table 2 below, the stratified results had limited statistical power, and most of the associations did not survive multiple testing correction, precluding definitive conclusions regarding sex-specific effects. Consequently, we unfortunately cannot reliably determine if there are meaningful sex differences in the intergenerational transmission patterns observed in our cohort.
**Table 2 Association between parental psychiatric conditions and offspring outcomes in the adjusted within-twin-family model, HR/OR (95% CI), sex-stratified analyses**

| **Exposure** | **Offspring outcomes** | **Father** | **Mother** | **Female** | **Male** |
| --- | --- | --- | --- | --- | --- |
| Any parental psychiatric conditions | Any psychiatric conditions | **1.36 (1.12, 1.64)** | 1.18 (1.00, 1.39) | 1.36 (1.09, 1.69) | 1.23 (1.01, 1.50) |
|  | Psychotic conditions | 1.33 (0.95, 1.85) | 1.13 (0.82, 1.55) | 1.77 (1.17, 2.67) | 1.36 (0.94, 1.97) |
|  | Neurodevelopmental conditions | 1.10 (0.81, 1.48) | 1.05 (0.78, 1.42) | 1.29 (0.93, 1.78) | 0.61 (0.42, 0.88) |
|  | Internalizing conditions | 1.20 (1.03, 1.38) | **1.27 (1.11, 1.46)** | 1.27 (1.06, 1.52) | **1.39 (1.19, 1.63)** |
|  | Substance use disorders | 1.32 (0.94, 1.86) | 1.18 (0.87, 1.60) | 1.35 (0.96, 1.89) | 1.07 (0.69, 1.64) |
|  | Externalizing behaviors | 1.26 (0.81, 1.95) | 0.79 (0.53, 1.17) | 1.19 (0.82, 1.73) | 0.40 (0.20, 0.80) |
|  | Suicide | 1.16 (0.84, 1.59) | 0.86 (0.62, 1.20) | 0.92 (0.63, 1.36) | 0.96 (0.66, 1.42) |
|  | Poor school performance | 1.22 (0.90, 1.65) | 1.15 (0.88, 1.49) | 1.21 (0.89, 1.63) | 1.23 (0.87, 1.75) |
|  | Long-term unemployment | 1.08 (0.80, 1.46) | 1.27 (0.99, 1.63) | 1.30 (0.94, 1.78) | 1.12 (0.80, 1.57) |
| Parental internalizing conditions | Any psychiatric conditions | 1.35 (1.06, 1.71) | 1.21 (1.01, 1.44) | 1.32 (1.03, 1.69) | 1.07 (0.85, 1.34) |
|  | Psychotic conditions | 1.53 (1.02, 2.29) | 1.12 (0.81, 1.55) | 1.65 (1.05, 2.59) | 1.41 (0.93, 2.15) |
|  | Neurodevelopmental conditions | 1.09 (0.75, 1.57) | 1.09 (0.81, 1.46) | 1.17 (0.83, 1.67) | 0.78 (0.53, 1.15) |
|  | Internalizing conditions | 1.04 (0.87, 1.25) | 1.24 (1.06, 1.44) | 1.14 (0.93, 1.41) | 1.28 (1.06, 1.55) |
|  | Substance use disorders | 1.61 (1.00, 2.59) | 1.04 (0.75, 1.43) | 1.54 (1.02, 2.32) | 0.72 (0.45, 1.17) |
|  | Externalizing behaviors | 1.27 (0.64, 2.52) | 0.65 (0.42, 1.01) | 1.01 (0.63, 1.61) | 0.48 (0.20, 1.14) |
|  | Suicide | 1.09 (0.72, 1.64) | 1.00 (0.69, 1.44) | 1.19 (0.75, 1.88) | 0.75 (0.48, 1.15) |
|  | Poor school performance | 1.76 (1.19, 2.60) | 1.20 (0.90, 1.60) | 1.52 (1.07, 2.17) | 1.41 (0.94, 2.13) |
|  | Long-term unemployment | 1.11 (0.75, 1.64) | 1.27 (0.96, 1.68) | 1.15 (0.79, 1.68) | 1.24 (0.82, 1.87) |
| Parental externalizing conditions | Any psychiatric conditions | 1.29 (1.04, 1.61) | 1.18 (0.91, 1.53) | 1.09 (0.79, 1.52) | 1.52 (1.14, 2.03) |
|  | Psychotic conditions | 0.97 (0.66, 1.43) | 0.62 (0.35, 1.09) | 1.34 (0.75, 2.41) | 0.58 (0.32, 1.06) |
|  | Neurodevelopmental conditions | 1.46 (1.02, 2.08) | 1.44 (0.91, 2.26) | 1.11 (0.67, 1.84) | 1.36 (0.76, 2.44) |
|  | Internalizing conditions | 1.00 (0.83, 1.20) | 0.82 (0.63, 1.06) | 0.84 (0.64, 1.09) | 1.22 (0.97, 1.54) |
|  | Substance use disorders | 1.34 (0.95, 1.91) | 0.99 (0.64, 1.52) | 1.10 (0.73, 1.65) | 1.68 (1.02, 2.77) |
|  | Externalizing behaviors | 1.27 (0.85, 1.89) | 1.06 (0.66, 1.70) | 1.46 (1.04, 2.05) | 0.48 (0.13, 1.76) |
|  | Suicide | 0.87 (0.52, 1.48) | 1.11 (0.69, 1.79) | 0.79 (0.44, 1.44) | 0.92 (0.46, 1.84) |
|  | Poor school performance | 1.16 (0.80, 1.68) | 0.71 (0.44, 1.14) | 1.00 (0.63, 1.60) | 1.31 (0.78, 2.22) |
|  | Long-term unemployment | 1.14 (0.78, 1.65) | 0.98 (0.61, 1.56) | 1.48 (0.94, 2.32) | 0.80 (0.45, 1.42) |

Note: Estimators shown in bold meet the criteria for Benjamini-Hochberg False Discovery Rate statistical significance (was conducted separately for parent and offspring). The estimator is the odds ratio for poor school performance and long-term unemployment.

3.11 Clarify the number of tests for FDR correction: (N = 18; 2 exposure categories × 9 offspring outcomes).

**Response:** We have now specified the number of tests used for FDR correction (N = 27; 3 exposure categories × 9 offspring outcomes) in both the manuscript text and the footnote of Figure 2.

On page 11 in the revised Method section, we now write:

*We corrected the between- and within-twin-family p-values for multiple testing (N = 9*3) with the* *Benjamini-Hochberg false discovery rate.*

On page 35 in the revised note of Figure 2, we now write:

*Estimators shown in bold meet the criteria for Benjamini-Hochberg False Discovery Rate statistical significance (was conducted separately for between-family model [N = 9*3] and within-twin family model [N = 9*3]).*

**Reviewer #4:** 
Thank you for inviting me to review this manuscript, which examines associations between parent psychiatric conditions and offspring outcomes. The study uses administrative data from Sweden and uses a clever "children-of-twins" design (essentially comparing cousins) to rule out genetic confounding. I think this is a very well-written manuscript, addressing an important question using sophisticated methods. The methods and findings are described very clearly (which is worth highlighting, given that the CoT design is quite complicated). I only had a few concerns: 
4.1 Offspring where between age 14 and 44 years old. It would be helpful to know if effects varied by age - 14 seems quite young for a psychiatric disorder diagnosed (and therefore captured in the registry data) as well as for some of the other outcomes, so it would be helpful to know if the analyses of younger outcome ages may have under-estimated effects. I understand that the analysis is underpowered as is, but even just descriptively presenting this comparison would be helpful.

**Response:** As noted in the General comment above, we conducted the analyses in an updated linkage (the minimum age is now 21, rather than 14), such that everyone has now arguably lived through the initial risk period for psychiatric conditions. Additionally, we have conducted exploratory analyses across three birth cohorts to address the reviewer’s concern. As shown in Table D in S1 Appendix (Sensitivity 4), these analyses suggest variability in effect sizes by follow-up age, though limited precision precludes definitive conclusions.

On page 12 in the revised Method section, we now write:

*Fourth, to examine whether the effects varied by follow-up age, we divided the study population into three birth cohorts: 1970-1980, 1980-1990, and 1990-2000, so that individuals were aged 41-51, 31-41, and 21-31 years, respectively, at the end of follow-up.*

On page 16 in the revised Result section, we now write:

*Fourth, when dividing the study population into three birth cohorts, most associations were nonsignificant due to wider confidence intervals, with the exception that significant associations were additionally observed between parental internalizing conditions and offspring suicide in the 1970-1980 cohort.*

4.2 If I understand correctly, the authors use lifetime diagnoses in the parent, which means that the parent could have been diagnosed before the child was born. This seems a bit problematic given that most theories of the intergenerational transmission of mental health problems would probably argue that child exposure to parental mental health problems is what matters (rather than, say, having a parent who was diagnosed with a mental health problem once before their child was born). If possible, it would be good to do sensitivity analyses taking this into account. This should also be pointed out as a limitation (if I'm correct).

**Response:** For the rationale to use parental lifetime diagnoses, please see our response to comment 2.2 above. To empirically assess the potential impact of exposure timing, we have now conducted a sensitivity analysis restricted to parents whose first psychiatric diagnosis occurred after childbirth. As shown in Table D in S1 Appendix (Sensitivity 2), all significant associations from the main analysis remained robust. Additionally, we observed significant associations between any parental psychiatric condition and offspring substance use disorders and poor school performance, as well as between parental externalizing conditions and offspring substance use disorders. We have incorporated this as a sensitivity analysis in the manuscript, and explicitly acknowledged using lifetime parental diagnoses as a limitation.

On page 12 in the revised Method section, we now write:

*Second, acknowledging that lifetime parental diagnoses include conditions predating childbirth - which may not fully align with developmental theories emphasizing the importance of the childrearing environment - we analyzed a subsample where we restricted the exposure to parental psychiatric conditions occurring after childbirth.*

On page 15 in the revised Result section, we now write:

*Second, when restricting the analysis to parents whose first psychiatric conditions were diagnosed after childbirth, the results were similar to the main analyses, with the exception that significant associations were additionally observed between any parental psychiatric conditions and offspring substance use disorders, poor school performance, and between parental externalizing conditions and offspring substance use disorders.*

On page 21 in the revised Limitation section, we now write:

*Ninth, our use of lifetime parental diagnoses did not ensure temporal precedence and includes conditions predating childbirth. While our sensitivity analyses restricting parental diagnoses occurring before the offspring's adolescence (before age 18) and to the post-childbirth period demonstrated robust effects, future studies could employ stricter temporal definitions for exposure.*

4.3 Why did the analysis focus on externalising and internalising, rather than also taking into account psychotic-like conditions (as was done for the offspring, and as was mention for a previous study in the Introduction)? I imagine this may have been due to power issues - it would be helpful to clarify.

**Response:** Please see our response 3.2 above.

4.4 It would be helpful to present (in the appendix) estimates of whether being a child of an MZ twin is predictive of any of the outcomes, just to pre-empt concerns that MZ twin offspring might be different from general population offspring.

**Response:** Existing literature comparing twins and singletons indicates that while minor early developmental differences exist, these largely attenuate by school age and are minimal in adulthood. Specifically, studies have reported that there are no significant differences between twins and singletons in personality traits [30], early social behavior [31], externalizing problems in early adolescence [32], or various health and lifestyle outcomes [33, 34]. Furthermore, a recent comprehensive study by Mönkediek et al. examining parenting styles found that results from twins appear to be generalizable to non-twin families, as most observed differences were attributable to age effects rather than twin status itself [35].

While we are unaware of studies that have directly compared outcomes for offspring of MZ twins versus singletons, the established pattern of minimal long-term differences between twins and singletons in adulthood provides indirect evidence suggesting their offspring are likely comparable. Nevertheless, we explicitly acknowledge the lack of direct empirical comparison in our study as a limitation. Our twin sample consists of survey respondents (which we used to determine their zygosity), and we lack access to a comparable non-twin survey sample with similar response characteristics, precluding direct assessment of offspring outcome differences. We have now acknowledged the generalizability of our findings as a limitation.

On page 20 in the revised Result section, we write:

*Third, the use of only children of Swedish MZ twins may limit the generalizability of our findings to the general non-twin population or to other countries.*

4.5 In the first section of the Results, please present the effective sample size in the text, or at least give an indication (e.g. report the range if the n varies across outcomes) - some of these n's drop to quite small numbers so I think it could be a bit misleading to only report those impressively-looking large numbers in the text.

**Response:** Please see our response to comment 3.3 above.

4.6 The Discussion focused on the null effects, but if I follow correctly, then within-family associations were significant for parental internalising problems and offspring educational attainment and unemployment, which is worth highlighting.

**Response:** Please see our response to comment 3.4 above.

4.7 The use of diagnoses is a strength, but it's also a limitation, because they capture only a subset of people with a mental health problem. This would be worth pointing out in the limitations section.

**Response:** As noted in our response to comments 2.4 and 3.8 above, the use of diagnoses likely captures cases identified in specialist care, and misses individuals treated exclusively in primary care or those who did not seek treatment. We have now added this limitation to the manuscript.

On page 20 in the revised Limitation section, we write:

*Fourth, we included only clinical diagnoses assigned in specialist care (i.e., we lacked information on individuals treated exclusively in primary care or those who did not seek treatment), so the associations are likely generalizable to cases identified in specialist care only.*

**References**

1.	Dagani J, Signorini G, Nielssen O, Bani M, Pastore A, Girolamo G, et al. Meta-analysis of the Interval between the Onset and Management of Bipolar Disorder. Can J Psychiatry. 2017;62(4):247–58. Epub 20160711. doi: 10.1177/0706743716656607. PubMed PMID: 27462036; PubMed Central PMCID: PMCPMC5407546.

2.	Hirschfeld RM, Lewis L, Vornik LA. Perceptions and impact of bipolar disorder: how far have we really come? Results of the national depressive and manic-depressive association 2000 survey of individuals with bipolar disorder. J Clin Psychiatry. 2003;64(2):161–74. PubMed PMID: 12633125.

3.	Krueger RF, Caspi A, Moffitt TE, Silva PA. The structure and stability of common mental disorders (DSM-III-R): a longitudinal-epidemiological study. J Abnorm Psychol. 1998;107(2):216–27. doi: 10.1037//0021-843x.107.2.216. PubMed PMID: 9604551.

4.	Pettersson E, Lichtenstein P, Larsson H, Song J, Agrawal A, Børglum AD, et al. Genetic influences on eight psychiatric disorders based on family data of 4 408 646 full and half-siblings, and genetic data of 333 748 cases and controls. Psychological Medicine. 2019;49(07):1166–73. doi: 10.1017/s0033291718002039.

5.	Zhou M, Lageborn CT, Sjölander A, Larsson H, D'Onofrio B, Landén M, et al. Psychiatric Diagnoses in Parents and Psychiatric, Behavioral, and Psychosocial Outcomes in Their Offspring: A Swedish Population-Based Register Study. Am J Psychiatry. 2024;181(8):761–73. doi: 10.1176/appi.ajp.20230353. PubMed PMID: 39086283.

6.	D'Onofrio BM, Turkheimer EN, Eaves LJ, Corey LA, Berg K, Solaas MH, et al. The role of the Children of Twins design in elucidating causal relations between parent characteristics and child outcomes. Journal of Child Psychology and Psychiatry. 2003;44(8):1130–44. doi: 10.1111/1469-7610.00196.

7.	D'Onofrio BM, Lahey BB, Turkheimer E, Lichtenstein P. Critical need for family-based, quasi-experimental designs in integrating genetic and social science research. Am J Public Health. 2013;103 Suppl 1(Suppl 1):S46–55. Epub 20130808. doi: 10.2105/AJPH.2013.301252. PubMed PMID: 23927516; PubMed Central PMCID: PMCPMC3778076.

8.	Kessing LV, Ziersen SC, Caspi A, Moffitt TE, Andersen PK. Lifetime Incidence of Treated Mental Health Disorders and Psychotropic Drug Prescriptions and Associated Socioeconomic Functioning. JAMA Psychiatry. 2023;80(10):1000–8. doi: 10.1001/jamapsychiatry.2023.2206. PubMed PMID: 37436730; PubMed Central PMCID: PMCPMC10339222.

9.	Porta M, Greenland S, Miguel Hernán, Santos Silva Id, M. Last J. A dictionary of epidemiology. 6th edition. New York: Oxford University Press; 2014.

10.	Everhov AH, Frisell T, Osooli M, Brooke HL, Carlsen HK, Modig K, et al. Diagnostic accuracy in the Swedish national patient register: a review including diagnoses in the outpatient register. Eur J Epidemiol. 2025;40(3):359–69. Epub 20250327. doi: 10.1007/s10654-025-01221-0. PubMed PMID: 40140143; PubMed Central PMCID: PMCPMC12137447.

11.	Sellgren C, Landen M, Lichtenstein P, Hultman CM, Langstrom N. Validity of bipolar disorder hospital discharge diagnoses: file review and multiple register linkage in Sweden. Acta Psychiatr Scand. 2011;124(6):447–53. Epub 20110813. doi: 10.1111/j.1600-0447.2011.01747.x. PubMed PMID: 21838734.

12.	Ruck C, Larsson KJ, Lind K, Perez-Vigil A, Isomura K, Sariaslan A, et al. Validity and reliability of chronic tic disorder and obsessive-compulsive disorder diagnoses in the Swedish National Patient Register. BMJ Open. 2015;5(6):e007520. Epub 20150622. doi: 10.1136/bmjopen-2014-007520. PubMed PMID: 26100027; PubMed Central PMCID: PMCPMC4480012.

13.	Krueger RF. The Structure of Common Mental Disorders. Archives of General Psychiatry. 1999;56(10):921–6. doi: 10.1001/archpsyc.56.10.921.

14.	Achenbach TM. Bottom-Up and Top-Down Paradigms for Psychopathology: A Half-Century Odyssey. Annu Rev Clin Psychol. 2020;16:1–24. doi: 10.1146/annurev-clinpsy-071119-115831. PubMed PMID: 32383999.

15.	Nordmo M, Kleppestø T, Sunde HF, Flatø M, Demange P, Torvik FA. The association between parental internalizing disorders and child school performance. npj Science of Learning. 2023;8(1). doi: 10.1038/s41539-023-00182-x.

16.	Nordsletten AE, Larsson H, Crowley JJ, Almqvist C, Lichtenstein P, Mataix-Cols D. Patterns of Nonrandom Mating Within and Across 11 Major Psychiatric Disorders. JAMA Psychiatry. 2016;73(4):354. doi: 10.1001/jamapsychiatry.2015.3192.

17.	Torvik FA, Eilertsen EM, Hannigan LJ, Cheesman R, Howe LJ, Magnus P, et al. Modeling assortative mating and genetic similarities between partners, siblings, and in-laws. Nat Commun. 2022;13(1):1108. Epub 20220301. doi: 10.1038/s41467-022-28774-y. PubMed PMID: 35233010; PubMed Central PMCID: PMCPMC8888605.

18.	Howe LJ, Lawson DJ, Davies NM, St Pourcain B, Lewis SJ, Davey Smith G, et al. Genetic evidence for assortative mating on alcohol consumption in the UK Biobank. Nat Commun. 2019;10(1):5039. Epub 20191119. doi: 10.1038/s41467-019-12424-x. PubMed PMID: 31745073; PubMed Central PMCID: PMCPMC6864067.

19.	Lykken DT, Tellegen A. Is human mating adventitious or the result of lawful choice? A twin study of mate selection. J Pers Soc Psychol. 1993;65(1):56–68. doi: 10.1037//0022-3514.65.1.56. PubMed PMID: 8355143.

20.	Markon KE, Krueger RF, Watson D. Delineating the structure of normal and abnormal personality: an integrative hierarchical approach. J Pers Soc Psychol. 2005;88(1):139–57. doi: 10.1037/0022-3514.88.1.139. PubMed PMID: 15631580; PubMed Central PMCID: PMCPMC2242353.

21.	Jami ES, Hammerschlag AR, Bartels M, Middeldorp CM. Parental characteristics and offspring mental health and related outcomes: a systematic review of genetically informative literature. Transl Psychiatry. 2021;11(1):197. Epub 20210401. doi: 10.1038/s41398-021-01300-2. PubMed PMID: 33795643; PubMed Central PMCID: PMCPMC8016911.

22.	Allegrini AG, Hannigan LJ, Frach L, Barkhuizen W, Baldwin JR, Andreassen OA, et al. Intergenerational transmission of polygenic predisposition for neuropsychiatric traits on emotional and behavioural difficulties in childhood. Nat Commun. 2025;16(1):2674. Epub 20250318. doi: 10.1038/s41467-025-57694-w. PubMed PMID: 40102402; PubMed Central PMCID: PMCPMC11920414.

23.	Pingault JB, Barkhuizen W, Wang B, Hannigan LJ, Eilertsen EM, Corfield E, et al. Genetic nurture versus genetic transmission of risk for ADHD traits in the Norwegian Mother, Father and Child Cohort Study. Mol Psychiatry. 2023;28(4):1731–8. Epub 20221116. doi: 10.1038/s41380-022-01863-6. PubMed PMID: 36385167; PubMed Central PMCID: PMCPMC10208953.

24.	Frach L, Barkhuizen W, Allegrini AG, Ask H, Hannigan LJ, Corfield EC, et al. Examining intergenerational risk factors for conduct problems using polygenic scores in the Norwegian Mother, Father and Child Cohort Study. Mol Psychiatry. 2024;29(4):951–61. Epub 20240116. doi: 10.1038/s41380-023-02383-7. PubMed PMID: 38225381; PubMed Central PMCID: PMCPMC11176059.

25.	Association of maternal polygenic risk scores for mental illness with perinatal risk factors for offspring mental illness.

26.	Birmaher B, Hafeman D, Merranko J, Zwicker A, Goldstein B, Goldstein T, et al. Role of Polygenic Risk Score in the Familial Transmission of Bipolar Disorder in Youth. JAMA Psychiatry. 2022;79(2):160–8. doi: 10.1001/jamapsychiatry.2021.3700. PubMed PMID: 34935868; PubMed Central PMCID: PMCPMC8696688.

27.	Cha J, Lee E, van Dijk M, Kim B, Kim G, Murphy E, et al. Polygenic scores for psychiatric traits mediate the impact of multigenerational history for depression on offspring psychopathology. Res Sq. 2024. Epub 20240717. doi: 10.21203/rs.3.rs-4264742/v1. PubMed PMID: 39070622; PubMed Central PMCID: PMCPMC11275997.

28.	McAdams TA, Neiderhiser JM, Rijsdijk FV, Narusyte J, Lichtenstein P, Eley TC. Accounting for genetic and environmental confounds in associations between parent and child characteristics: A systematic review of children-of-twins studies. Psychological Bulletin. 2014;140(4):1138–73. doi: 10.1037/a0036416.

29.	Zhou M, Larsson H, D’Onofrio BM, Landén M, Lichtenstein P, Pettersson E. Association between the childhood rearing environment and general and specific psychopathology factors in middle adulthood: a Swedish National High-Risk Home-Reared versus Adopted-Away Sibling Comparison Study. Molecular Psychiatry. 2025. doi: 10.1038/s41380-025-02979-1.

30.	Johnson W, Krueger RF, Bouchard TJ, McGue M. The Personalities of Twins: Just Ordinary Folks. Twin Research. 2012;5(2):125–31. doi: 10.1375/twin.5.2.125.

31.	A Comparison of the Early Social Behavior of Twins and Singletons. 1981.

32.	Robbers SC, Bartels M, van Oort FV, van Beijsterveldt CE, van der Ende J, Verhulst FC, et al. A twin-singleton comparison of developmental trajectories of externalizing and internalizing problems in 6- to 12-year-old children. Twin Res Hum Genet. 2010;13(1):79–87. doi: 10.1375/twin.13.1.79. PubMed PMID: 20158310.

33.	Andrew T, Hart DJ, Snieder H, de Lange M, Spector TD, MacGregor AJ. Are twins and singletons comparable? A study of disease-related and lifestyle characteristics in adult women. Twin Res. 2001;4(6):464–77. doi: 10.1375/1369052012803. PubMed PMID: 11780939.

34.	de Geus EJC, Posthuma D, Ijzerman RG, Boomsma DI. Comparing Blood Pressure of Twins and Their Singleton Siblings: Being a Twin Does Not Affect Adult Blood Pressure. Twin Research. 2012;4(5):385–91. doi: 10.1375/twin.4.5.385.

35.	Mönkediek B, Schulz W, Eichhorn H, Diewald M. Is there something special about twin families? A comparison of parenting styles in twin and non-twin families. Soc Sci Res. 2020;90:102441. Epub 20200712. doi: 10.1016/j.ssresearch.2020.102441. PubMed PMID: 32825925.

**Comments from editors**

*We ask every co-author listed on the manuscript to fill in a contributing author statement, making sure to declare all competing interests. If any of the co-authors have not filled in the statement, we will remind them to do so when the paper is revised. If all statements are not completed in a timely fashion this could hold up the re-review process. If new competing interests are declared later in the revision process, this may also hold up the submission. Should there be a problem getting one of your co-authors to fill in a statement we will be in contact. Please do not add or remove authors without first discussing this with the handling editor. You can see our competing interests policy here: <https://eur01.safelinks.protection.outlook.com/?url=http%3A%2F%2Fjournals.plos.org%2Fplosmedicine%2Fs%2Fcompeting-interests&data=05%7C02%7Cmengping.zhou%40ki.se%7Ceb1a6c6708924d15a93908dd9e9dd4fd%7Cbff7eef1cf4b4f32be3da1dda043c05d%7C0%7C0%7C638841122927763357%7CUnknown%7CTWFpbGZsb3d8eyJFbXB0eU1hcGkiOnRydWUsIlYiOiIwLjAuMDAwMCIsIlAiOiJXaW4zMiIsIkFOIjoiTWFpbCIsIldUIjoyfQ%3D%3D%7C0%7C%7C%7C&sdata=91%2FaiZr3NIs1nYSIDGcbeAbO4LsKS2O4to8mFMA3x%2FY%3D&reserved=0>.
**Response:** We ensure that all co-authors complete the statement and declare all competing interests.

*Please upload any figures associated with your paper as individual TIF or EPS files with 300dpi resolution at resubmission; please read our figure guidelines for more information on our requirements: <https://eur01.safelinks.protection.outlook.com/?url=http%3A%2F%2Fjournals.plos.org%2Fplosmedicine%2Fs%2Ffigures&data=05%7C02%7Cmengping.zhou%40ki.se%7Ceb1a6c6708924d15a93908dd9e9dd4fd%7Cbff7eef1cf4b4f32be3da1dda043c05d%7C0%7C0%7C638841122927775980%7CUnknown%7CTWFpbGZsb3d8eyJFbXB0eU1hcGkiOnRydWUsIlYiOiIwLjAuMDAwMCIsIlAiOiJXaW4zMiIsIkFOIjoiTWFpbCIsIldUIjoyfQ%3D%3D%7C0%7C%7C%7C&sdata=4Iizf0W3uSbsdEW9OaNsSeaprGY%2FLlnEdt5xG1tG%2BLk%3D&reserved=0>. While revising your submission, please upload your figure files to the PACE digital diagnostic tool, <https://eur01.safelinks.protection.outlook.com/?url=https%3A%2F%2Fpacev2.apexcovantage.com%2F&data=05%7C02%7Cmengping.zhou%40ki.se%7Ceb1a6c6708924d15a93908dd9e9dd4fd%7Cbff7eef1cf4b4f32be3da1dda043c05d%7C0%7C0%7C638841122927787628%7CUnknown%7CTWFpbGZsb3d8eyJFbXB0eU1hcGkiOnRydWUsIlYiOiIwLjAuMDAwMCIsIlAiOiJXaW4zMiIsIkFOIjoiTWFpbCIsIldUIjoyfQ%3D%3D%7C0%7C%7C%7C&sdata=xAIdf5EEP7TorGBWT4B%2FP3OzjKoEETHvyntjiVnO7Y0%3D&reserved=0>. PACE helps ensure that figures meet PLOS requirements. To use PACE, you must first register as a user. Then, login and navigate to the UPLOAD tab, where you will find detailed instructions on how to use the tool. If you encounter any issues or have any questions when using PACE, please email us at [PLOSMedicine@plos.org](mailto:PLOSMedicine@plos.org).
**Response:** We confirmed that the submission of all the figures meets all the requirements mentioned.

*Please ensure that the paper adheres to the PLOS Data Availability Policy (see <https://eur01.safelinks.protection.outlook.com/?url=http%3A%2F%2Fjournals.plos.org%2Fplosmedicine%2Fs%2Fdata-availability&data=05%7C02%7Cmengping.zhou%40ki.se%7Ceb1a6c6708924d15a93908dd9e9dd4fd%7Cbff7eef1cf4b4f32be3da1dda043c05d%7C0%7C0%7C638841122927799141%7CUnknown%7CTWFpbGZsb3d8eyJFbXB0eU1hcGkiOnRydWUsIlYiOiIwLjAuMDAwMCIsIlAiOiJXaW4zMiIsIkFOIjoiTWFpbCIsIldUIjoyfQ%3D%3D%7C0%7C%7C%7C&sdata=J5ThuHmXZr3eiX4EFkdcZ53CFnNAWCLOccWvVphtyMw%3D&reserved=0)>, which requires that all data underlying the study's findings be provided in a repository or as Supporting Information. For data residing with a third party, authors are required to provide instructions with contact information (web or email address) for obtaining the data. Please note that a study author cannot be the contact person for the data. PLOS journals do not allow statements supported by "data not shown" or "unpublished results." For such statements, authors must provide supporting data or cite public sources that include it.
**Response:** We confirmed that the study adheres to the PLOS Data Availability Policy.

*We expect all researchers with submissions to PLOS in which author-generated code underpins the findings in the manuscript to make all author-generated code available without restrictions upon publication of the work. In cases where code is central to the manuscript, we may require the code to be made available as a condition of publication. Authors are responsible for ensuring that the code is reusable and well documented. Please make any custom code available, either as part of your data deposition or as a supplementary file. Please add a sentence to your data availability statement regarding any code used in the study, e.g. "The code used in the analysis is available from Github [URL] and archived in Zenodo [DOI link]" Please review our guidelines at <https://eur01.safelinks.protection.outlook.com/?url=https%3A%2F%2Fjournals.plos.org%2Fplosmedicine%2Fs%2Fmaterials-software-and-code-sharing&data=05%7C02%7Cmengping.zhou%40ki.se%7Ceb1a6c6708924d15a93908dd9e9dd4fd%7Cbff7eef1cf4b4f32be3da1dda043c05d%7C0%7C0%7C638841122927810753%7CUnknown%7CTWFpbGZsb3d8eyJFbXB0eU1hcGkiOnRydWUsIlYiOiIwLjAuMDAwMCIsIlAiOiJXaW4zMiIsIkFOIjoiTWFpbCIsIldUIjoyfQ%3D%3D%7C0%7C%7C%7C&sdata=SwxLxJCkdAHB0GN690qI0fKKFKm13YBvaZJiZZP5ZLQ%3D&reserved=0> and ensure that your code is shared in a way that follows best practice and facilitates reproducibility and reuse. Because Github depositions can be readily changed or deleted, we encourage you to make a permanent DOI'd copy (e.g. in Zenodo) and provide the URL. 
**Response:** We confirmed that all the code will be available should the paper be accepted for publication.

*Please ensure that the study is reported according to the STROBE guideline and include the completed STROBE checklist as Supporting Information. When completing the checklist, please use section and paragraph numbers, rather than page numbers. Please add the following statement, or similar, to the Methods: "This study is reported as per STROBE guideline (S1 Checklist)."
**Response:** We confirmed that the study has been reported following the STROBE guidelines, and we have included the completed STROBE checklist.

FORMATTING - GENERAL
*Abstract: Please structure your abstract using the PLOS Medicine headings (Background, Methods and Findings, Conclusions). Please combine the Methods and Findings sections into one section.
**Response:** We confirmed that the abstract was structured using the PLOS Medicine headings.

*At this stage, we ask that you include a short, non-technical Author Summary of your research to make findings accessible to a wide audience that includes both scientists and non-scientists. The Author Summary should immediately follow the Abstract in your revised manuscript. This text is subject to editorial change and should be distinct from the scientific abstract. Ideally each sub-heading should contain 2-3 single sentence, concise bullet points containing the most salient points from your study. In the final bullet point of 'What Do These Findings Mean?', please include the main limitations of the study in non-technical language. Please see our author guidelines for more information: <https://eur01.safelinks.protection.outlook.com/?url=https%3A%2F%2Fjournals.plos.org%2Fplosmedicine%2Fs%2Frevising-your-manuscript%23loc-author-summary&data=05%7C02%7Cmengping.zhou%40ki.se%7Ceb1a6c6708924d15a93908dd9e9dd4fd%7Cbff7eef1cf4b4f32be3da1dda043c05d%7C0%7C0%7C638841122927822298%7CUnknown%7CTWFpbGZsb3d8eyJFbXB0eU1hcGkiOnRydWUsIlYiOiIwLjAuMDAwMCIsIlAiOiJXaW4zMiIsIkFOIjoiTWFpbCIsIldUIjoyfQ%3D%3D%7C0%7C%7C%7C&sdata=y7pte%2BpXFEk8U98ENkKvnm7lbN4qOb7lLP8ZsPSpf3I%3D&reserved=0>.
**Response:** We have now included the Author Summary in the revised manuscript.

*Please express the main results with 95% CIs as well as p values. When reporting p values please report as p<0.001 and where higher as the exact p value p=0.002, for example. Throughout, suggest reporting statistical information as follows to improve clarity for the reader "22% (95% CI [13%,28%]; p</=)". Please be sure to define all numerical values at first use.
**Response:** We have now reported both 95% CIs and p values.

*Please include page numbers and line numbers in the manuscript file. Use continuous line numbers (do not restart the numbering on each page). 
**Response:** We have now added page numbers and line numbers.

*Please cite the reference numbers in square brackets. Citations should precede punctuation.
**Response:** We have now adapted the citation format to meet the requirements.

FIGURES AND TABLES
*Please provide titles and legends for all figures and tables (including those in Supporting Information files).
**Response:** We confirmed that titles and legends for all figures and tables are provided.

*Please define all abbreviations used in each figure/table (including those in Supporting Information files).
**Response:** We confirmed that all abbreviations have been defined.

*Please consider avoiding the use of red and green in order to make your figure more accessible to those with color blindness.
**Response:** We have now redrawn the figure without using red and green.

SUPPLEMENTARY MATERIAL
*Please note that supplementary material will be posted as supplied by the authors. Therefore, please amend it according to the relevant comments outlined here.
*Please cite your Supporting Information as outlined here: <https://eur01.safelinks.protection.outlook.com/?url=https%3A%2F%2Fjournals.plos.org%2Fplosmedicine%2Fs%2Fsupporting-information&data=05%7C02%7Cmengping.zhou%40ki.se%7Ceb1a6c6708924d15a93908dd9e9dd4fd%7Cbff7eef1cf4b4f32be3da1dda043c05d%7C0%7C0%7C638841122927833544%7CUnknown%7CTWFpbGZsb3d8eyJFbXB0eU1hcGkiOnRydWUsIlYiOiIwLjAuMDAwMCIsIlAiOiJXaW4zMiIsIkFOIjoiTWFpbCIsIldUIjoyfQ%3D%3D%7C0%7C%7C%7C&sdata=eCS6M3Y%2BOHnINygDBCwx2NIXaPt%2F%2BzWX47eV6IYfVrw%3D&reserved=0>
**Response:** We have now adapted the supplementary material to meet the requirements.

REFERENCES
*PLOS uses the numbered citation (citation-sequence) method and first six authors, et al.
*Please ensure that journal name abbreviations match those found in the National Center for Biotechnology Information (NCBI) databases (<https://eur01.safelinks.protection.outlook.com/?url=http%3A%2F%2Fwww.ncbi.nlm.nih.gov%2Fnlmcatalog%2Fjournals&data=05%7C02%7Cmengping.zhou%40ki.se%7Ceb1a6c6708924d15a93908dd9e9dd4fd%7Cbff7eef1cf4b4f32be3da1dda043c05d%7C0%7C0%7C638841122927844901%7CUnknown%7CTWFpbGZsb3d8eyJFbXB0eU1hcGkiOnRydWUsIlYiOiIwLjAuMDAwMCIsIlAiOiJXaW4zMiIsIkFOIjoiTWFpbCIsIldUIjoyfQ%3D%3D%7C0%7C%7C%7C&sdata=GAkV2lq%2FYvYUWN6LubUVNvUv%2B0DY297qP%2B1ce9BALJE%3D&reserved=0>), and are appropriately formatted and capitalised.
*Where website addresses are cited, please include the complete URL and specify the date of access (e.g. [accessed: 12/06/2023]).
*Please also see <https://eur01.safelinks.protection.outlook.com/?url=https%3A%2F%2Fjournals.plos.org%2Fplosmedicine%2Fs%2Fsubmission-guidelines%23loc-references&data=05%7C02%7Cmengping.zhou%40ki.se%7Ceb1a6c6708924d15a93908dd9e9dd4fd%7Cbff7eef1cf4b4f32be3da1dda043c05d%7C0%7C0%7C638841122927856334%7CUnknown%7CTWFpbGZsb3d8eyJFbXB0eU1hcGkiOnRydWUsIlYiOiIwLjAuMDAwMCIsIlAiOiJXaW4zMiIsIkFOIjoiTWFpbCIsIldUIjoyfQ%3D%3D%7C0%7C%7C%7C&sdata=s%2FqDMBz51S5b4bK%2BQXGvBYd%2Fn7NYlmhntmZX1R4EGyo%3D&reserved=0> for further details on reference formatting.
**Response:** We confirmed that references were cited in the correct style and format.

OBSERVATIONAL STUDIES
*Abstract: Please include the study design, population and setting, number of participants, years during which the study took place (enrollment and follow up), length of follow up, and main outcome measures.
**Response:** We confirmed that the Abstract meets all the requirements mentioned.

*Please ensure that the study is reported according to the STROBE (or appropriate STOBE extension) guideline (available from: <https://eur01.safelinks.protection.outlook.com/?url=https%3A%2F%2Fwww.equator-network.org%2Freporting-guidelines%2Fstrobe&data=05%7C02%7Cmengping.zhou%40ki.se%7Ceb1a6c6708924d15a93908dd9e9dd4fd%7Cbff7eef1cf4b4f32be3da1dda043c05d%7C0%7C0%7C638841122927867601%7CUnknown%7CTWFpbGZsb3d8eyJFbXB0eU1hcGkiOnRydWUsIlYiOiIwLjAuMDAwMCIsIlAiOiJXaW4zMiIsIkFOIjoiTWFpbCIsIldUIjoyfQ%3D%3D%7C0%7C%7C%7C&sdata=jZCMHZvMCi2OBCcdqUndI2jqcFIBIwSa6HjnSM%2BTrAA%3D&reserved=0)> and include the completed STROBE (or STROBE extension) checklist as Supporting Information. Please add the following statement, or similar, to the Methods: "This study is reported as per the Strengthening the Reporting of Observational Studies in Epidemiology (STROBE) guideline (S1 Checklist)." When completing the checklist, please use section and paragraph numbers, rather than page numbers. 
**Response:** We confirmed that the study has been reported following the STROBE guidelines, and we have included the completed STROBE checklist.

*For all observational studies, in the manuscript text, please indicate: (1) the specific hypotheses you intended to test, (2) the analytical methods by which you planned to test them, (3) the analyses you actually performed, and (4) when reported analyses differ from those that were planned, transparent explanations for differences that affect the reliability of the study's results. If a reported analysis was performed based on an interesting but unanticipated pattern in the data, please be clear that the analysis was data driven. 
**Response:** We confirmed that the manuscript meets all the requirements mentioned.

*Please state in the Methods section whether the study had a prospective protocol or analysis plan. If a prospective analysis plan (from your funding proposal, IRB or other ethics committee submission, study protocol, or other planning document written before analyzing the data) was used in designing the study, please include the relevant document(s) with your revised manuscript as a Supporting Information file to be published alongside your study and cite it in the Methods section. A legend for this file should be included at the end of your manuscript. If no such document exists, please make sure that the Methods section transparently describes when analyses were planned, and when/why any data-driven changes to analyses took place. Changes in the analysis, including those made in response to peer review comments, should be identified as such in the Methods section of the paper, with rationale.

**Response:** We confirmed that all analyses were planned before conducting any analyses and obtaining the results, and that no data-driven analyses were performed at any stage. As detailed in the General comment above, we have reanalyzed all models using updated registry data with extended follow-up through December 31, 2020.
