# Supplementary material for: Association between parental psychiatric conditions and offspring psychiatric, behavioral, and psychosocial outcomes: A Swedish population-based children-of-monozygotic twins study
Source: PLoS Med. 2025 Oct 21;22(10):e1004784. doi: 10.1371/journal.pmed.1004784 (PMC12571287; doi:10.1371/journal.pmed.1004784)
Supplement: Attachment — Submitted filename: Response letter_to editor.docx [file pmed.1004784.s008.docx]

**Comments from Editor**

1) Include the clarification regarding the existence of an analysis plan in the methods, and include the study proposal as a supplement.

**Response:** Thank you for the suggestion! We have now added this clarification as recommended and included the study proposal as a supplement.

On page 12 in the revised Statistical Analyses section, we now write:

*Original analyses, using data up to December 2013, were planned verbally between May and July 2023, with analyses carried out between September 2023 and September 2024. In response to reviewers’ comments, we re-ran the analyses with data up to December 2020 (see S2 Appendix for study proposal).*

2) Thank you for including the limitations in your author summary, but this bullet point is quite long. Could you change this into 2 bullet points? A) ‘the children-of-MZ-twins design does not control for unmeasured factors, meaning residual confounding factors could still be present’ and b) ‘This study is based on clinical diagnoses assigned in specialist care, and therefore likely not generalizable to cases treated exclusively in primary care, or to individuals who did not seek treatment’.

**Response:** Thank you for the valuable suggestion. We have now divided this limitation into two bullet points. For the first point, we retained the phrase “not shared by cousins” because the children-of-MZ-twins design can control for unmeasured factors that are shared by cousins. Removing this phrase could therefore lead to misunderstanding.

On page 4 in the revised ‘What Do These Findings Mean?’, we now write:

*• The children-of-MZ-twins design cannot control for unmeasured factors not shared by cousins, meaning residual confounding factors could still be present.*

*• This study is based on clinical diagnoses assigned in specialist care, and therefore likely not generalizable to cases treated exclusively in primary care, or to individuals who did not seek treatment.*

3) Could you shorten your abstract? it is currently over the maximum of 500 words allowed. I would suggest removing several of the explanation within parentheses (see below).

**Response:** Thank you for the helpful suggestion. We have removed several explanatory phrases within parentheses as recommended. The abstract now contains 498 words, which is within the journal’s word limit.

**Comments from Reviewers**

All reviewer comments have been fully addressed in prior revisions, and the reviewers are satisfied with the responses. No further comments remain at this stage.
